# Dengue Vector Dynamics (*Aedes aegypti*) Influenced by Climate and Social Factors in Ecuador: Implications for Targeted Control

**DOI:** 10.1371/journal.pone.0078263

**Published:** 2013-11-12

**Authors:** Anna M. Stewart Ibarra, Sadie J. Ryan, Efrain Beltrán, Raúl Mejía, Mercy Silva, Ángel Muñoz

**Affiliations:** 1 Center for Global Health and Translational Sciences, State University of New York Upstate Medical University, Syracuse, New York, United States of America; 2 Department of Environmental and Forest Biology, State University of New York College of Environmental Science and Forestry, Syracuse, New York, United States of America; 3 National Service for the Control of Vector-Borne Diseases, Ministry of Health, Machala, Ecuador; 4 National Institute of Meteorology and Hydrology, Guayaquil, Ecuador; 5 Department of Agriculture, Engineering, and Science, School of Life Sciences, University of KwaZulu-Natal, Pietermaritzburg, South Africa; 6 International Institute of Climate and Society, Columbia University, New York, New York, United States of America; 7 Centro de Modelado Científico, Universidad del Zulia, Maracaibo, Venezuela; Louisiana State University, United States of America

## Abstract

**Background:**

Dengue fever, a mosquito-borne viral disease, is now the fastest spreading tropical disease globally. Previous studies indicate that climate and human behavior interact to influence dengue virus and vector (*Aedes aegypti*) population dynamics; however, the relative effects of these variables depends on local ecology and social context. We investigated the roles of climate and socio-ecological factors on *Ae. aegypti* population dynamics in Machala, a city in southern coastal Ecuador where dengue is hyper-endemic.

**Methods/Principal findings:**

We studied two proximate urban localities where we monitored weekly *Ae. aegypti* oviposition activity (Nov. 2010-June 2011), conducted seasonal pupal surveys, and surveyed household to identify dengue risk factors. The results of this study provide evidence that *Ae. aegypti* population dynamics are influenced by social risk factors that vary by season and lagged climate variables that vary by locality. Best-fit models to predict the presence of *Ae. aegypti* pupae included parameters for household water storage practices, access to piped water, the number of households per property, condition of the house and patio, and knowledge and perceptions of dengue. Rainfall and minimum temperature were significant predictors of oviposition activity, although the effect of rainfall varied by locality due to differences in types of water storage containers.

**Conclusions:**

These results indicate the potential to reduce the burden of dengue in this region by conducting focused vector control interventions that target high-risk households and containers in each season and by developing predictive models using climate and non-climate information. These findings provide the region's public health sector with key information for conducting time and location-specific vector control campaigns, and highlight the importance of local socio-ecological studies to understand dengue dynamics. See Text S1 for an executive summary in Spanish.

## Introduction

Dengue is the most widely distributed vector-borne disease in Latin America and the Caribbean, reported by 40 out of 45 countries and territories from 2000 to 2010 [1]. Until a dengue vaccine becomes available, reducing *Aedes aegypti* (dengue mosquito vector) populations remains the primary means of preventing outbreaks. Identifying effective vector control interventions requires a better understanding of the drivers of *Ae. aegypti* population dynamics at a scale of analysis that matches the spatial and temporal scale of the intervention, often seasonal interventions at the household or community level.

Studies have shown that climate variability influences dengue transmission and *Ae. aegypti* population dynamics in the Americas, indicating the potential to develop public health interventions using climate information [2]–[6]. Warmer air and water temperatures can increase rates of larval development [7]–[9], adult biting rates, gonotrophic development [10], [11], and the extrinsic incubation period of the virus in the mosquito [12]. Studies have shown that *Ae. aegypti* are positively associated with areas with high relative humidity and high vegetation, ideal conditions for adult mosquito refugia [13]. The effect of rainfall is more complex. Rainfall events can increase mosquito abundance by increasing the availability of mosquito juvenile habitat (e.g., containers in the patio with standing water). However, heavy rainfall events can decrease mosquito abundance by flushing larvae from containers [14] and drought events can increase mosquito abundance by increasing household water storage [15].

The effect of climate on *Ae. aegypti* abundance may vary within a region due, in part, to a suite of social factors that interact with climate to influence vector dynamics. For example, studies from drier and wetter regions of Puerto Rico found that *Ae. aegpyti* densities were positively correlated with rainfall only [3], correlated with temperature only [5], and not correlated with either temperature or rainfall [16]. This variation may be attributed, in part, to factors such as water storage practices, people's knowledge and risk perception, and housing conditions that affect the density of containers with water [13], [17]–[21]. In regions where dengue transmission is associated with seasonal climate variability, the suite of social factors associated with dengue risk may vary seasonally; however, few studies have tested this hypothesis. Identifying the most important household-level risk factors in each season would provide information for decision makers to fine-tune vector control interventions.

In this study, we therefore investigated the seasonally differentiated household risk factors and climate triggers influencing *Ae. aegypti* abundance in Machala, a city in southern coastal Ecuador where dengue is hyper-endemic. A previous study in this region found that dengue transmission is highly seasonal and outbreaks are driven in part by climate, *Ae. aegypti* abundance, and the number of dengue serotypes circulating [22]. Using a multi-model selection process, we tested whether household risk factors for the presence of *Ae. aegypti* pupae varied during different seasons; we identified the most important lagged climate variables influencing *Ae. aegypti* population dynamics; and we tested whether significant climate factors varied between the study localities. We conducted field studies in two urban sites in Machala: a central area (CA) with access to public services and a newer peripheral area (PA) with limited service access, where we (1) monitored weekly *Ae. aegypti* oviposition activity and local climate (Nov. 2010-June 2011), (2) conducted seasonal pupal surveys to identify key container types and to measure *Ae. aegypti* pupae presence and abundance, and (3) conducted household surveys to identify risk factors. The results of this study provide the region's public health sector with important information for conducting time and location-specific vector control campaigns, with the goal of reducing vector densities below an epidemic threshold.

## Materials and Methods

### Ethics statement

The investigation protocol was developed in collaboration with the National Service for the Control of Vector Borne Diseases of the Ministry of Health of Ecuador and was reviewed and approved by the Institutional Review Board of Syracuse University. Heads of households aged 18 years or older signed an informed consent form before participating in the study. All field and laboratory components of this research were conducted in collaboration with technicians from the Ecuadorian National Service for the Control of Vector Borne Diseases (El Oro Province).

### Study area

Dengue re-emerged in Ecuador in the late 1980s, and is now hyper-endemic in the coastal lowland region, where all four serotypes have co-circulated since the year 2000 [23], [24].

This study took place in the southern coastal city of Machala, El Oro province (pop. 241,606), where the average annual dengue incidence across all serotypes was 28.5 cases per 10,000 population (2003–2011 average). No information was available regarding the incidence or health effects of each serotype.

**Figure 1:**
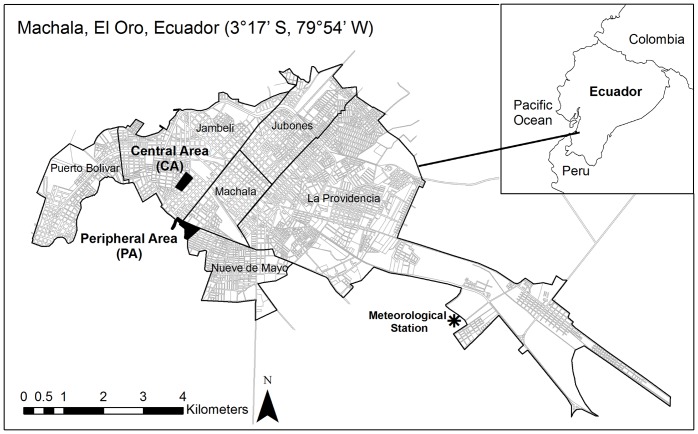
Map of study localities. A map of the districts of the city of Machala, El Oro Province, Ecuador, indicating the location of the study areas and the meteorological station.

Six months after the most severe dengue epidemic on record in the province, we investigated two nearby urban areas (½ km apart) in southern Machala where the epidemic began: (1) PA (population 1269) comprising two adjacent communities, Primero de Enero and Heroes de Jambeli, and (2) CA (population 904) comprised of one community named Veinte-Cinco de Diciembre (Fig. 1). During the epidemic, forty cases of dengue were reported from the PA and sixteen cases were reported from the CA. The CA was located in the southern-central sector of the city and surrounded by dense urban housing. The streets were paved and most households had access to municipal garbage collection, sewerage, and a constant supply of piped water inside the home. The PA was located at the southernmost edge of the city, bordered by mixed commercial and residential buildings just to the north, and mangroves and abandoned shrimp ponds to the south. Streets were unpaved, and significantly fewer households had access to sewerage, garbage collection and piped water inside the home. Many PA households (38%) reported daily or weekly interruptions in the piped water supply, and as a result, a greater proportion of PA households store water (54% in PA; 23% in CA).

### Climate variables

**Figure 2:**
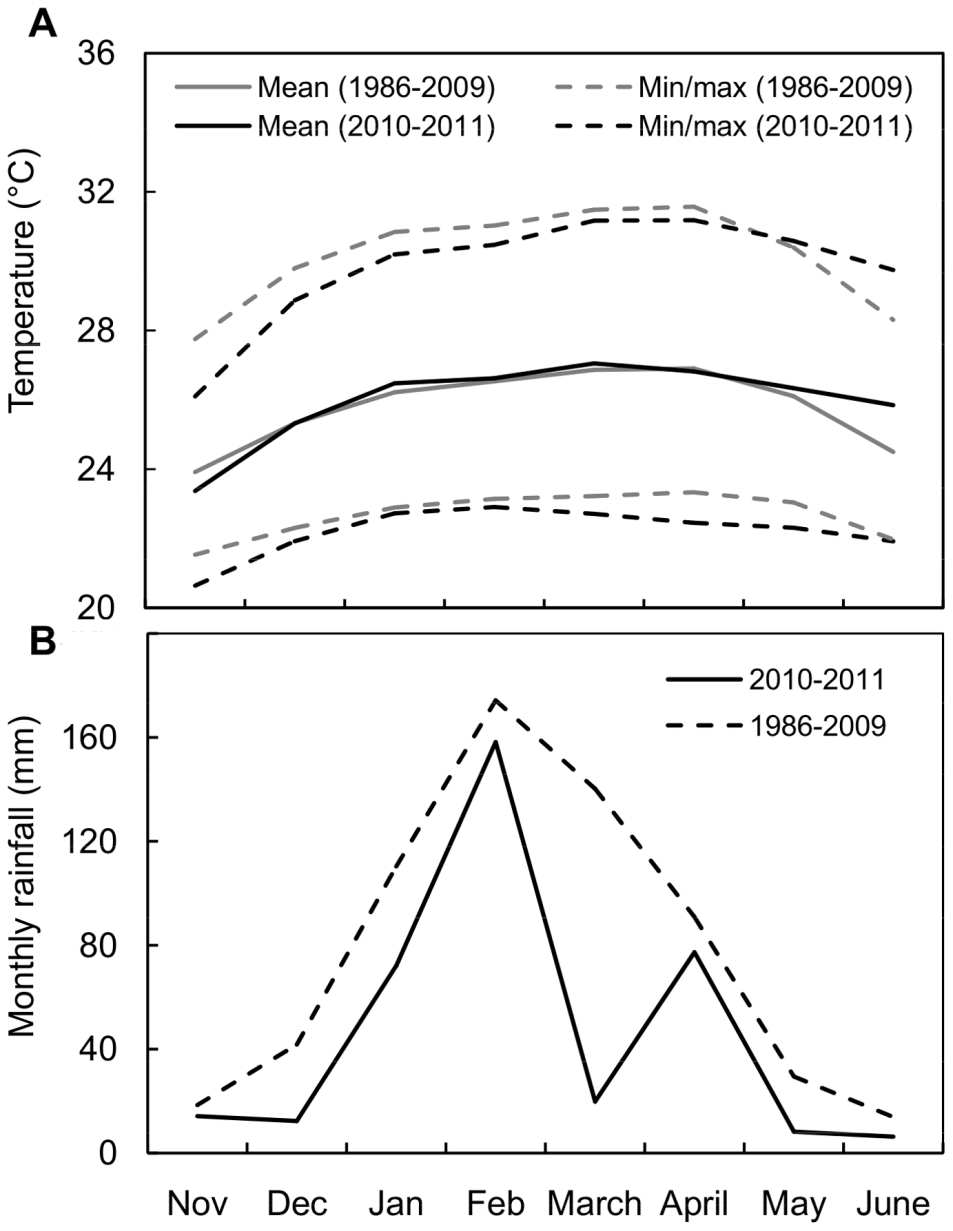
Climate in Machala, Ecuador. The climatology of Machala (November to June, 1986–2009 average) compared to the weather during the study period for (A) temperature and (B) monthly rainfall.

Peak dengue transmission occurs during the hot, rainy season from December to May (mean rainfall  = 3.3 mm/day, mean temperature  = 26.4°C), and transmission persists at low levels during the cooler, dry season the rest of the year (mean rainfall  = 0.44 mm/day, mean temperature  = 23.6°C) (Fig. 2). The El Niño-Southern Oscillation (ENSO) causes significant year-to-year variability in local climate [25], [26], leading to dengue outbreaks during warm, rainy El Niño events [22]. Daily meteorological data (rainfall, relative humidity, mean/min/max air temperature) during the study period were provided by the Granja Santa Ines weather station located in Machala and operated by the National Institute of Meteorology and Hydrology (INAMHI) of Ecuador, and we calculated weekly averages for each variable over the study period (3°17′16” S, 79°54′5” W, 5 m.a.s.l.). This weather station provided the only source of publically available climate data for the city, and the most complete climatological time series for the coastal region of El Oro province.

The weather station was located approximately 8 km from the study sites (Fig. 1), close enough to capture the general behavior of the weather variables, as confirmed in the following analyses. A cluster investigation of monthly precipitation data for 121 INAMHI meteorology stations, from 1971–2010, revealed homogeneity in seasonal climate patterns from the stations located in this region of Ecuador. Additionally, we analyzed the wind circulation patterns from the National Centers for Environmental Prediction-National Center for Atmospheric Research (NCEP-NCAR) Reanalysis Project version 2 and from 13 years of simulation using the Weather Research and Forecasting Model (WRF) (source: International Research Institute Data Library of Columbia University), and found that temperature and moisture advection are also spatially homogeneous over Machala [27]. Although the station data is obviously not suitable for characterizing the fine-spatial scale within the urban environment (<0.5 km, e.g., the local noise component), the background weather signal was successfully captured by the station. This permitted us to develop statistical models to predict *Ae. aegypti* oviposition dynamics, as discussed in the statistical analysis section below.

### Weekly ovitrap sampling

We monitored weekly *Ae. aegypti* oviposition activity from November 2010 to June 2011 (32 weeks) by installing modified CDC ovitraps at households at each study locality (80 traps total, 40 per locale; approx. 6.3 ovitraps/ha) [28]–[31]. In the CA, ovitraps were distributed uniformly across nine city blocks that spanned a 0.4 by 0.2 km rectangular area. In the PA, ovitraps were distributed uniformly across sixteen small city blocks in an L-shaped area at the city margin, 0.5 by 0.1 km and 0.01 by 0.2 km. Ovitraps have been used to provide a cost-effective and sensitive means of monitoring the presence of *Ae. aegypti* and seasonal dynamics, although it should be noted that ovitraps do not provide a direct measure of adult abundance and egg count data may be influenced by the availability of alternative oviposition containers in the environment [32]. Each ovitrap consisted of two 1-liter black plastic buckets with a white cotton cloth oviposition substrate [30]. Buckets contained 100% and 10% aqueaous infusions of grass (*Panicum maximum*), created by fermenting fresh mature leaves for 15 days, to attract gravid female *Ae. aegypti* [31]. Traps were uniformly distributed across the study areas and were installed in the peridomestic area. Cloths were collected from traps once per week and taken to the laboratory, where eggs were counted and reared to fourth instar larvae, at which stage they could be identified as *Ae. aegypti* or non-*Ae. aegypti* under a stereoscope microscope. Larvae were reared by placing each cloth in a 1-liter container with approximately 500 ml water and a fine mesh cover at ambient air temperatures. Larvae were fed larval shrimp food on days 1, 3, and 5; water in the containers was changed on days 3 and 5. Larvae that hatched from cloths with a high density of eggs (e.g., more than 100) were separated into multiple containers to avoid overcrowding. Ovitrap results were recorded as the total number of *Ae.aegypti* eggs/ovitrap/week, and we calculated the average eggs/ovitrap/week for each site and for both sites combined. Any households that dropped out of the study were replaced within one week by the closest household on the same block willing to participate.

### Seasonal pupal surveys

Using standard pupal survey methods, we conducted three surveys in the same households that had ovitraps to measure the presence and abundance of *Ae.aegypti* pupae and to identify characteristics of the most productive containers [33]. Based on well-characterized seasonal patterns in climate and dengue transmission, survey sample dates were selected to capture *Ae. aegypti* behaviors during the pre-rainy season (November 2010), the rainy season (February 2011) and the post-rainy season (June 2011) [22]. Pupae were collected by straining the water from all containers (up to 250 gallon water tanks), re-suspending the sieved contents in a white enamel tray, and pipetting the pupae into vials. Any larvae that were collected in the sediment were discarded and were not returned to containers. Containers that could not be emptied (e.g., cisterns) were visually inspected and all visible pupae were collected using a mesh sweep net (15 cm diameter, 20 cm depth). We were able to inspect all cisterns and 69% of elevated water tanks, which are used for water storage by 66% of the population in the PA and 95% in the CA. All pupae were collected and raised to adults in the laboratory for species identification. We recorded the descriptive information about each container including container type, use, source of water, and location inside or outside the home (See Table S1 for descriptors and the correlation matrix of characteristics of containers positive for *Ae. aegypti* pupae).

### Household surveys

We surveyed residents from 79 of the 80 households that had ovitraps (March – May, 2011) to identify household factors associated with the presence of *Ae. aegypti* pupae. Descriptions of the survey variables are presented in Table 1 (See Table S2 for coding of knowledge variables). Before conducting the survey, we conducted focus groups with community members from the study area to identify locally relevant social and environmental risk factors, and modified the survey instrument accordingly. The condition of the patio, condition of the home, and proportion of the patio area that was shaded (described in Table 1) were recorded using the Premise Condition Index (PCI) methodology [34], which has been shown to be an effective predictor for presence of *Ae. aegypti* in other regions [34]–[36]. The three variables that make up the PCI were tested individually in the model along with all other variables rather than as a composite index.

**Table 1:** Socio-ecological factors hypothesized to influence presence of *Aedes aegypti* in households.

Parameter	Parameter value (percentage of households or mean ± SE)
**1. Demographic**	
Female head of household	27.8% (22/79)
Education level of the head of household	Post secondary education 27.6% (21/76); Secondary education or less 72.4% (55/76)
Head of household is currently employed or seeking work	Working or seeking work 88.6% (70/79); Retired, disabled, receives a pension, housewife 11.4% (9/79)
Average age of the family	Young family (average age <35) 63.3% (50/79); Older family (average age 35–64) 31.6% (25/79); Old family (average age 65+) 5.1% (4/79)
Number of people in the household	4.3±0.2 (range 1–10, *n* = 79)
People per room in the household	1.38±0.09 (range 0.33–5, *n* = 79)
Number of independent households residing on the property	1 household 67.1% (53/79); 2 households 19% (15/79); 3 or more households 13.9% (11/79)
Renters present on the property	13.9% (11/79)
**2. Water access & storage**	
Piped water infrastructure	Piped water inside the household 69.6% (55/79); Piped water on the property outside the household 30.3% (24/79)
Access to piped water	Constant access to piped water 76.6% (59/77); Weekly or daily water interruptions 23.4% (18/77)
Water storage: No Cist/ET & do store	No cistern or elevated tank (Cist/ET) and do store water 19.5% (15/77)
Water storage: Cist/ET & don't store	Do have Cist/ET and do not store water 62.3% (48/77)
Water storage: Cist/ET & do store	Do have Cist/ET and do store water 18.2% (14/77)
**3. Knowledge & perceptions**	
Knowledge of mosquito habitat	Dengue mosquito juveniles are found in clean water, standing water, or containers 78.5% (62/79).
Knowledge of dengue transmission	Dengue is transmitted by mosquitoes 73% (58/79)
Dengue is a problem	Yes 91.1% (72/79); No or don't know 8.9% (7/79)
Dengue is preventable	Yes 91.1% (72/79); No or don't know 8.9% (7/79)
Dengue severity	Dengue is a severe disease 57% (45/79); Dengue is mild, moderate, other or don't know 43% (34/79)
**4. Housing condition**	
Density of trees in the patio	0.05±0.01 trees per meter of patio (range 0–0.5, *n* = 75)
High, medium, or low proportion of the patio area shaded	High shade 11.4% (9/79); Medium shade 43% (34/79); Low shade 45.6% (36/79)
Abandoned lots bordering the property	53.8% (42/78)
Patio condition	Bad 34.2% (27/79); Normal 48.1 (38/79); Good 17.7% (14/79)
House condition	Bad 21.5% (17/79); Normal 34.2% (27/79); Good 44.3% (35/79)
Screens on windows and doors	No screens 55.7% (44/79); Some or all screens present 44.3% (35/79)
Log of area of the patio (sq. meters)	1.62±0.07 (range 0.3–3.38, *n* = 75)

### Statistical analysis

#### Household risk factors

We hypothesized that the presence of *Ae. aegypti* pupae was related to one or more of the household risk factors listed in Table 1. Using an information-theoretic approach, described below, we assessed the influence of these factors on the presence of *Ae. aegypti* during each survey period, to identify the most important factors in each season [37]. This approach is appropriate for this type of study, where there are a large number of potential socio-ecological explanatory variables, and in which we have considerable a *priori* knowledge about the system.

Four hypotheses were developed to predict the presence of *Ae. aegypti* pupae based on the literature and our experience working in this region. Each hypothesis was described as a suite of socio-ecological variables (described in Table 1): 1. human demographic characteristics, 2.knowledge and perceptions, 3.water access and storage, and 4. housing condition (See correlation matrices for variable subsets in Table S3). Logistic regression models for each hypothesis were selected for each of the three survey periods (pre-rainy season, rainy season, post-rainy season). Model selection was computed using “glmulti,” an R package for multimodel selection [38], and final models were evaluated using GLM in R. We used glmulti to test all possible unique models from our suites of variables and ranked them based on Akaike's Information Criterion (AIC) modified for small sample sizes (AICc). AICc is a measure of the relative goodness-of-fit of the model (Eq. 1), where k is the number of variables in the model, *L* is the maximum value of the likelihood function of the estimated model, and *n* is the sample size [39]. AICc modifies the AIC to include a greater penalty for extra parameters. When comparing a set of candidate models, smaller values of AICc indicate a model that fits the data better.



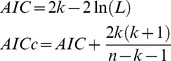
Eq. 1


For each suite of variables in our hypotheses, a best model was obtained, using the threshold criteria of AICc ≤2. When using glmulti, the same model will always be selected as the best model, given the same initial parameters, unlike step-wise model selection procedures. The variables selected in the best models for each hypothesis were then included into one combined model for each survey period. We then conducted multimodel selection on these pooled variables, to derive the best-fit model overall; again, using glmulti package in R. A dummy variable for the study localities was included in the pooled model selection to test for differences among localities not captured by the explanatory variables. Adjusted odds ratios (OR) and 95% confidence intervals (CI) were calculated for all variables included in subset and final models, and presented in Table 2 and Table S4.

**Table 2:** Parameters in the top-ranked logistic models to predict the presence of *Aedes aegypti* in each season.

Parameters	β estimate	SE	OR	Lower 95% CI	Upper 95% CI	*P* value
**Pre rainy season (n = 68)**						
Intercept	−8.662	2.59				<0.01
Renters present on the property	−5.481	2.654	0	0	0.76	0.04
3 or more households	5.331	1.965	206.72	4.39	9729.02	<0.01
Have cist/ET & also store water	4.668	1.628	106.52	4.38	2589.59	<0.01
Piped water inside the home	5.04	1.983	154.54	3.17	7529.57	0.01
Bad patio condition	2.627	1.234	13.83	1.23	155.29	0.03
Old family	2.312	1.878	10.1	0.25	400.48	0.22
**Rainy season (n = 75)**						
Intercept	−0.92	0.75				0.22
Have cist/ET & also store water	1.65	0.79	5.22	1.11	24.49	0.04
Knowledge of mosquito habitat	−1.86	0.79	0.16	0.03	0.72	0.02
Bad patio condition	1.27	0.62	3.56	1.05	12.08	0.04
Bad house condition	1.42	0.71	4.15	1.02	16.81	0.046
Older family	−1.29	0.78	0.28	0.06	1.26	0.10
Location: central neighborhood	1.02	0.65	2.77	0.77	9.88	0.12
**Post rainy season (n = 75)**						
Intercept	3.17	1.641				0.05
One household	−3.183	1.157	0.04	0	0.4	<0.01
Have cist/ET & also store water	3.661	1.113	38.89	4.39	344.81	<0.01
Constant access to piped water	−3.059	1.106	0.05	0.01	0.41	<0.01
Dengue is a problem	−2.905	1.58	0.05	0	1.21	0.07

Slope coefficient estimates and adjusted odds ratios (OR) with 95% confidence intervals (CI) for parameters included in the top-ranked logistic regression models for each season.

#### Climate and Aedes aegypti dynamics

We developed statistical models to identify the most important lagged local climate variables influencing *Ae. aegypti* population dynamics and to test whether significant climate factors varied between the study localities. We modeled log_10_-transformed ovitrap data from the CA, PA, and both localities combined (eggs/ovitrap/week) as a function of climate using a general linear model. We identified the most important lags to test in the model by assessing significant correlations between ovitrap data and climate variables at lags from 0 to 19 weeks, a similar time frame as the lags tested in a recent study of dengue and climate in the same region (See raw climate and ovitrap data in Fig. S1) [22]. We used these parameters to derive a best-fit model using glmulti in R [38]. A dummy variable for study locality was included in the best-fit model for both localities combined to capture confounding factors (e.g., socioeconomic differences, microclimate variability).

## Results

### Key containers for *Aedes aegypti*

We inspected a total of 2,492 containers with water and collected 809 *Ae. aegypti* pupae in the three surveys. Pupae were concentrated in few key premises, with 11% of all households containing 81.7% of pupae collected during the rainy season. Pupae were further concentrated in the post-rainy season, with 5% of households containing 80% of pupae collected (See Table S5 for pupal indices and proportion of positive household and containers by locale and season).

Differences in pupal indices between locations and seasons were observed, however, these were not statistically significant (*P*≥0.05). Pupal indices were highest during the rainy season and were higher in the CA in all seasons except for the post-rainy season (Table S5). From rainy to post-rainy seasons, CA pupal indices declined by 79%, and PA pupal indices declined by 22%; the proportion of CA households with pupae declined from 35% to 11%, whereas the proportion of PA remained constant (23%). PA pupal indices declined less than CA indices, due to a higher proportion of *Ae. aegypti* pupae found in domestic-use containers in the PA, which likely sustained the mosquito population in the post-rainy season.

**Figure 3:**
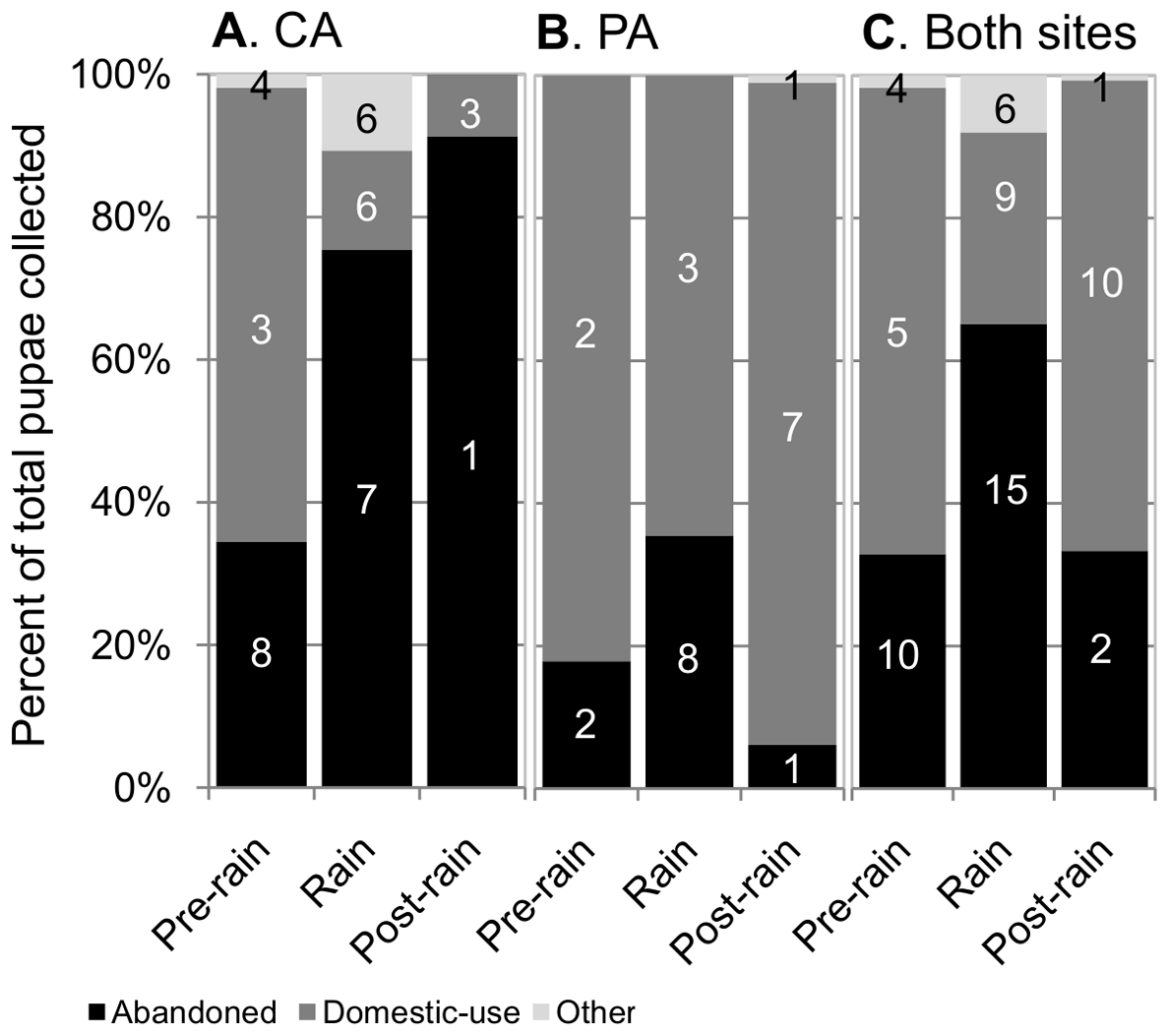
*Aedes aegypti* pupae per container type by location and season. Percentage of all pupae collected from abandoned, domestic-use, and other types of containers (i.e., decorative, animal drinking water) in pupae surveys conducted during pre-rainy, rainy, and post-rainy seasons in the (A) central study area (CA), (B) peripheral study area (PA), and (C) both localities combined in Machala, Ecuador.

We collected the majority of pupae from abandoned containers during the rainy season (65% of pupae) and from domestic-use containers during the drier pre- and post-rainy seasons (65.4% and 66% of pupae, respectively) (Fig. 3). Abandoned containers with pupae tended to be rain-filled and located outdoors (e.g., tires, empty food containers), whereas domestic-use containers with pupae tended to be tap-water filled and located either indoors or outdoors (e.g., barrels), as shown by significant bivariate correlations (P≤0.05, Table S1). On average across the three surveys, the majority of pupae in the CA were found in abandoned containers (58% of all pupae), and the majority of pupae in the PA were found in domestic-use containers (79% of all pupae). However, due to the limited number of replicates (one survey per site per season) we were unable to test the statistical significance of the proportion of pupae per container use (e.g., abandoned, domestic use) reported by study site and season in Figure 3.

Barrels were the most productive container, producing 69% and 63% of all pupae in the pre- and post-rainy seasons, and 38% of pupae in the post-rainy season (See Table S6 for pupae production by container type and season). No pupae were collected from elevated water tanks and only one pupa was collected from a cistern.

### Household risk factors

The best-fit models of household risk factors for presence of *Ae. aegypti* pupae varied by season, and included variables for water storage practices, access to potable water, the number of independent households per property, the condition of the house and patio, and knowledge and perceptions of dengue. We found that in all three season, several models were within our threshold criterion of **Δ**AICc≤2 of the top model fit, but with fairly consistent significant variables, suggesting that there are multiple interacting important socio-ecological factors at play, which emerge in the competing top models. Best-fit model parameters and related statistics are given in Table 2 (best model correlation matrices are given in Table S7, best model subsets given in Table S4, top ranked models given in Table S8). Study location was not significant, suggesting that the best-fit sets of parameters were important predictors of the presence of *Ae. aegypti* pupae in each season regardless of location.

In all seasons, we found that households that stored water and also had cisterns or elevated water tanks (referred to below as the water storage parameter) had greater odds of being positive for *Ae. aegypti* pupae than households that either (1) did not store water, or (2) did store water but did not have a cistern or elevated water tank. Other significant parameters in the best-fit model for the pre-rainy season included properties shared by three or more households, access to piped water inside the home, the absence of renters, and bad patio condition. No other parameters were significant in any of the competing top models for the pre-rainy season (Table S8). Significant factors in the best-fit model for the rainy season included water storage, bad house condition, bad patio condition, and a lack of knowledge of *Ae. aegypti* juvenile habitats. The presence of an older family (average age 35–64) was an additional significant risk factor in one other competing model. Significant parameters in the best-fit post-rainy season model included water storage, daily or weekly interruptions in the piped water supply, and properties shared by two or more households. The perception that dengue is not a problem was also a significant risk factor in one other competing model.

### Climate and *Aedes aegypti* dynamics

We collected a total of 237,120 eggs in ovitraps; *Ae. aegypti* made up 97.6% of 4^th^ instar larvae reared from eggs. During the study period (Nov. 2010 to June 2011), cumulative rainfall was 40% below annual average, and minimum and maximum temperatures were slightly below average, due to a cool, dry La Niña episode from July 2010 to April 2011. As with pupal indices, we observed a seasonal temporal trend in *Ae. aegypti* oviposition, with a peak in mid-February, several weeks after the beginning of the rainy season. The two localities exhibited similar seasonal dynamics (Pearson Correlation: *r* = 0.79, *P*≤0.01), although significantly more *Ae. aegypti* eggs/ovitrap/week were collected from the CA (115.3±13.5) than the PA (63.9±6.1) (Welch's two sample t-test, t = −3.48, d.f.  = 61.1, *P*<0.001). To evaluate whether pupal surveys influenced egg counts, we compared egg counts the week before and 1 and 2 weeks after pupal surveys in households positive for *Ae. aegypti* pupae using an exact binomial test. We found that there was no effect. One week after pupal surveys, we found that egg counts increased or remained the same in 28 households and decreased in 23 households across the three surveys (P≥0.05). Egg counts two weeks later increased or remained the same in 22 households and decreased in 29 households (P≥0.05).

We found that all climate variables were significantly correlated with ovitrap data across a range of lags (See lagged correlations for combined data in Fig. S2. The following lags were most significant for both localities combined and for CA only: relative humidity at 6 weeks, mean/min/max temperature at 6 weeks, and log_10_ precipitation at 3 weeks (*P*≤0.05). The most significant lags for the PA were relative humidity at 6 weeks, mean and max temperature at 6 weeks, minimum temperature at 9 weeks, and log_10_ precipitation at 2 weeks (*P*≤0.05).

**Figure 4:**
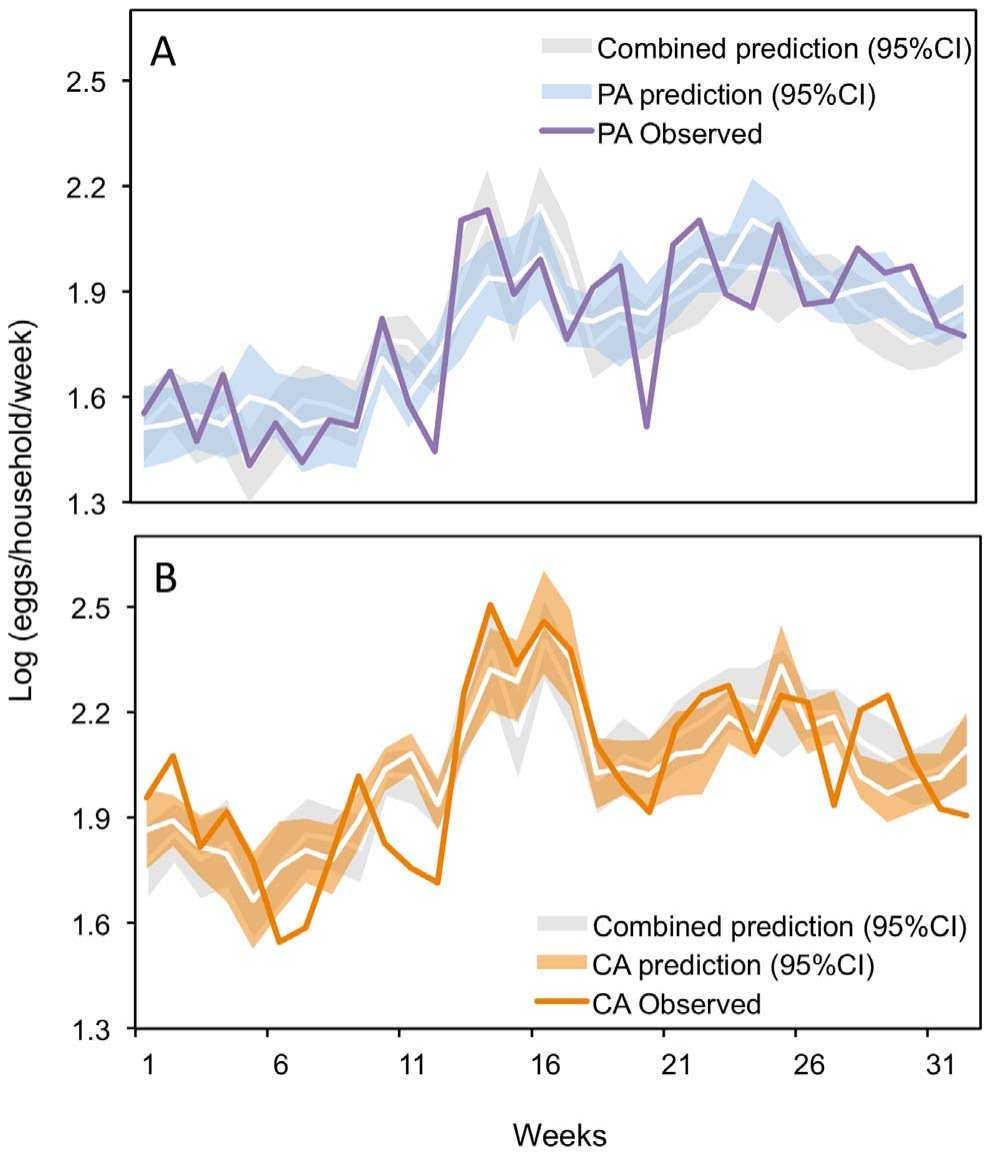
*Aedes aegypti* oviposition dynamics predicted by lagged local climate. Time series of observed and predicted (95% CI) log eggs/ovitrap/week over the study period (Nov. 2010 to June 2011) from the best-fit models for the (A) peripheral area (PA) and (B) central area (CA).

The best-fit model for the combined data (both sites) explained 69% of the variance in ovitrap data (adj. R^2^). All lagged climate variables and the dummy variable for study site were significant in the model (Fig. 4, Fig. S3A, Table 3). In the best-fit model, egg counts were positively associated with rainfall, minimum temperature, maximum temperature, and CA location (dummy variable), and negatively associated with relative humidity, mean temperature, and PA location. Rainfall and minimum temperature were the only climate parameters that were also significant in the other three top competing models, evidence that these parameters were important climate predictors (Table S9).

**Table 3:** Local climate parameters and lags in the best-fit model for *Aedes aegypti* ovitrap abundance data for both localities combined, for the central area (CA) and peripheral area (PA).

Parameters	β estimate	SE	Lower 95% CI	Upper 95% CI	*P* value
**Both localities (adj. R^2^ = 69%)**					
Intercept	2.69	1.80	−0.92	6.31	0.141
Log10(rainfall) (3 week lag)	0.27	0.07	0.13	0.40	<0.01
Minimum temperature (6 week lag)	0.25	0.09	0.07	0.42	<0.01
Relative humidity (6 week lag)	−0.03	0.01	−0.05	0.00	0.034
Maximum temperature (6 week lag)	0.17	0.08	0.02	0.32	0.028
Mean temperature (6 week lag)	−0.36	0.16	−0.68	−0.04	0.027
Locality (1 = CA, 0 = PA)	0.26	0.04	0.19	0.34	<0.01
**CA (adj. R^2^ = 58%)**					
Intercept	−0.89	0.66	−2.24	0.47	0.190
Log10(rainfall) (3 week lag)	0.38	0.09	0.19	0.58	<0.01
Minimum temperature (6 week lag)	0.13	0.03	0.07	0.19	<0.01
**PA (adj. R^2^ = 61%)**					
Intercept	0.93	1.81	−2.77	4.64	0.611
Log10(rainfall) (2 week lag)	0.14	0.09	−0.04	0.32	0.125
Minimum temperature (9 week lag)	0.10	0.04	0.02	0.19	0.021
Relative humidity (6 week lag)	−0.02	0.01	−0.04	0.01	0.136

Best-fit models developed for each site (CA and PA) show that the significant climate variables varied by site (Fig. 4, Fig. S3BC, Table 3). The best-fit model for ovitrap data from the CA explained 58% of the variance; rainfall and minimum temperature were positively associated with egg counts and statistically significant in the model. Mean temperature and rainfall were significant predictors in the other competing model. The best-fit model for ovitrap data from the PA explained 61% of the variance; minimum temperature was the only significant parameter. Rainfall and relative humidity were significant in one other competing model; however, minimum temperature was the only parameter that was significant in all seven competing models.

Figure 4 compares observed egg count data to predictions from the combined model and the locality-specific models. This figure shows that the combined model and locality-specific models predicted similar trends at each site; however, the locality-specific models were able to capture a higher degree of local variability.

## Discussion

**Figure 5:**
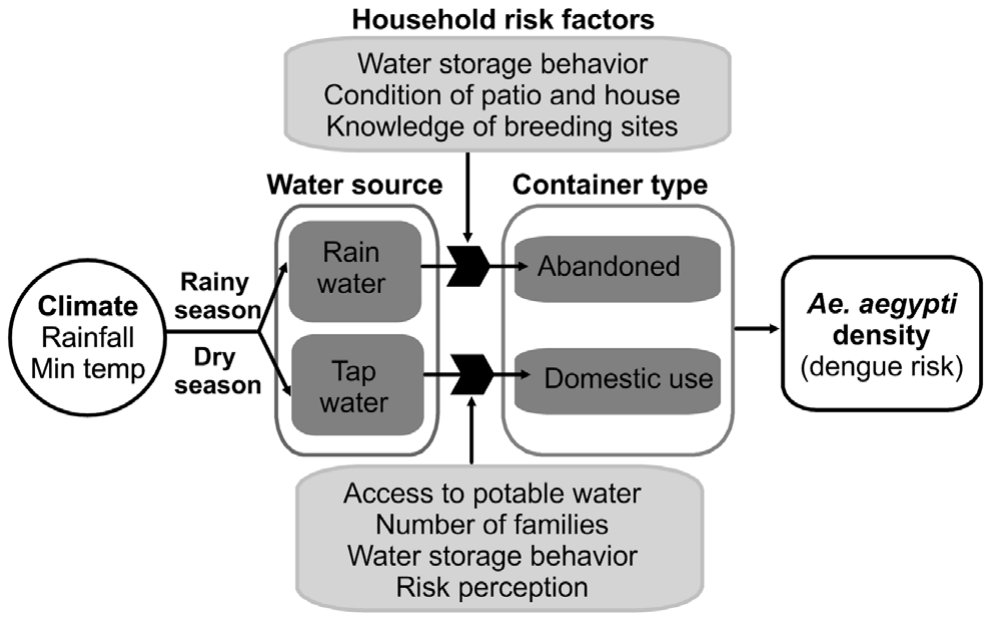
Climatic and social factors interact to influence seasonal dengue risk. A synthesis of the important socio-ecological predictors for the presence of *Aedes aegypti* during rainy and post-rainy (dry) seasons in Machala, Ecuador.

The results of this study provide evidence that *Ae. aegypti* population dynamics are influenced by social risk factors that vary by season and lagged climate variables that vary by locality (Fig. 5). We present an initial description of the characteristics of key larval containers in this region, demonstrating a high degree of variability at a fine-spatial scale within the urban environment (<0.5 km). Our findings highlight the importance of conducting local longitudinal field studies at multiple spatial scales that are relevant to vector control decisions. These results indicate the potential to reduce the burden of dengue in this region by developing predictive models using climate and non-climate information [40], and by conducting focused vector control interventions that target high-risk households and containers in each season [41].

Contrary to expectation, pupal indices and the results of the ovitrap-climate model suggest that *Ae. aegypti* were more abundant in the more urbanized and developed CA than the PA. During the rainy season, three times more pupae were collected from the CA than the PA. Both sites had an equal proportion of containers that were positive for pupae (3%); however, CA households had 75% more containers per household (Table S5). Why did households in the CA have more water-filled containers if they had greater access to piped water and garbage collection services? One possible answer is that the infrastructure improvements in the CA were relatively recent. The sociocultural risk factors identified in this study (e.g., human behavior and demographics) may not have changed during this short time frame. To better address this question, we would ideally sample across a gradient of urbanization, including communities that have had improved urban infrastructure for many years. It should be noted that the differences in pupal indices between the CA and PA were not statistically significant and ovitrap data do not provide a direct measure of adult abundance. Additionally, vector abundance is only one of many factors that influence dengue risk, including the immune status of the population, the viruses circulating in the region, and barriers to contact with mosquitoes (e.g., window screens, air conditioning) [42].

We found that *Ae. aegypti* abundance was positively associated with all lagged climate parameters; however, rainfall and minimum temperature were the most important predictors, findings that agree with a prior analysis of 16 years of dengue and climate data from the same province [22] and other studies in the Americas [4], [43]–[46]. Minimum temperature was a significant predictor at both localities. Studies have shown that warmer air and water temperatures decrease the extrinsic incubation period [12], increase dengue virus titers in mosquitoes [12], [47], shorten the gonotrophic cycle [10], and decrease development rates of immature mosquitoes [7]–[9]. Studies in Thailand found that minimum air temperature was the most important climate factor influencing *Ae. aegypti* biting rates [11]. Daily temperature fluctuations also influence dengue transmission dynamics by influencing larval development and survival, adult female reproduction, and vector susceptibility to viral infection [48]–[50].

The results of this and other studies indicate that minimum temperature is a key regulating climate parameter for dengue. In our study, the average monthly minimum temperature ranged from 20.6°C to 22.9°C, the lower end of the optimal temperature range for endemic dengue transmission (20°C−30°C) [9]. It is possible that a gradual increase in minimum temperature due to climate warming may increase dengue transmission in this region by increasing the number of days per year of optimal transmission.

At the city-level (i.e., both sites combined), we found that *Ae. aegypti* oviposition dynamics were positively associated with rainfall. *Ae. aegypti* were most abundant during the rainy season likely due to the presence of abandoned, rain-filled containers (Fig. 3, Table S5). As a result, bad condition of the home and patio (e.g., poorly maintained, untidy) were key risk factors during the pre-rain and rainy seasons. During the drier seasons, the *Ae. aegypti* population was likely sustained at a lower density in domestic-use (e.g., water storage) containers filled with tap water. Accordingly, interruptions in the piped water supply was a risk factor in the post-rainy season, and households that reported water supply interruptions had 70% more domestic-use containers per household than households with constant water supply (6.3 versus 3.7 containers/household).

Poor access to the water supply was likely exacerbated by below-average rainfall during the study period (Fig. 2). Previous studies have shown that rainfall shortages can increase dengue risk in areas where people store water [13], [15], [51]. Households that shared their property with other independent households were also at greater risk pre- and post-rainy season. Sharing a common space, such as a patio, may affect people's water storage practices (e.g., frequency of cleaning, emptying, and covering containers), thereby creating mosquito habitat. Previous studies similarly found that population density, housing patterns, and density of containers with water were associated with greater risk of dengue fever and mosquito abundance [20], [46], [52]–[55].

At the neighborhood level, we found that the impact of rainfall varied by locality due to differences in the dominant types of containers with larval *Ae. aegypti*. Rainfall was not a significant predictor of *Ae. aegypti* population dynamics in the PA, likely due to the predominance of tap-water filled containers. This finding suggests that educational messages should be developed at the neighborhood-level to focus on high-risk container types, avoiding ecological fallacy. This finding also highlights the importance of incorporating social data with climate information when developing spatially explicit dengue prediction models.

Sampling within one year limited our ability to discern whether the household risk factors, key containers, and climate drivers described in this study are typical of the average season or are anomalous findings associated with drier than average conditions. Previous studies have shown significant spatial and seasonal variation in *Ae. aegypti* abundance and key larval habitats [21], [41]. This high degree of variability indicates the importance of conducting additional surveys across a greater number of neighborhoods and over longer periods of time to be able to characterize *Ae. aegypti* population dynamics. Conducting a comparative study across a gradient of dengue transmission intensity (e.g., high to low incidence) would also improve our understanding of the roles of climate variability and mosquito population dynamics on dengue transmission. While our model selection procedure revealed several key household risk factors,, the competing models suggest that other factors may also contribute to the variance found at the household level. This indicates a need for further investigation to refine and improve our ability to inform local-level public health interventions.

Although we found that locality was not a significant predictor of the presence of pupae and that the climate in this region is spatially homogeneous, this study could be improved with information on microclimate variability between the two sites, shown to be an important predictor of *Ae. aegypti* dynamics in previous studies [13]. It is possible that temperature and relative humidity varied between the two sites due to proximity to mangroves and other vegetation, abandoned shrimp ponds that filled with rain during the rainy season, and other differences in the urban environment such as pavement. To explore the effect of microclimate on dengue transmission, INAMHI and the Ministry of Health of Ecuador have collaborated to install six additional weather stations throughout Machala in 2013.

### Policy implications

*Targeted interventions.* Our findings indicate that locally developed rapid household surveys could be used to identify high risk households to be targeted for vector control in each season [34]–[36]. Rapid surveys are especially important in areas where *Ae. aegypti* are concentrated in a small proportion of households, and Ministry of Health technicians are not able inspect 100% of households due to resource constraints. In this region, for example, multi-household properties could be targeted for vector control during the pre-rain and post-rainy seasons, and households with bad patio or bad house condition could be targeted in the rainy season.

Our findings support many previous studies which show that pupal surveys are an effective means of identifying the most productive containers to be targeted for vector control, a strategy hypothesized to be an effective means of preventing dengue outbreaks [21], [33], [41], [56]. In future studies, a larger sample of households would allow us to identify the key larval habitats in each season with greater confidence and investigate possible cryptic breeding sites, such as subterranean refugia [57]–[59]. Although cisterns and elevated water tanks do not appear to be key larval habitats, we were unable to inspect 31% of water tanks. Alternative sampling strategies that have been validated for large water storage containers should also be tested [60]. Using this information, vector control interventions can be developed to reduce pupal indices below the epidemic threshold, estimated to range from 0.26 to 1.05 pupae/person [61]. For example, if barrels were eliminated as mosquito habitat from the PA (e.g., through the use of covers or larvicide), pupal indices would fall below the epidemic threshold, declining from 0.34 to 0.031 pre-rainy season, 0.89 to 0.20 rainy season, and 0.69 to 0.07 post-rainy season. Although it is unlikely that 100% of barrels could be eliminated, this example highlights the epidemiological importance of focusing on the most productive container types. Longitudinal studies should be conducted to evaluate whether adult female *Ae. aegypti* are reduced when the most productive containers and high-risk households are targeted, or whether mosquitoes are able to sustain their populations by modifying their breeding behaviors.

*Water storage practices:* Improving piped water infrastructure has the potential to reduce dengue risk in the urban periphery. Other studies also found that access to piped water and water supply interruptions were important risk factors for the presence of *Ae. aegypti* and dengue [13], [17], [52], [53]. However, a study in Vietnam showed that improvements in water infrastructure did not change household water storage practices or *Ae. aegypti* larval indices [62]. For this reason, infrastructure improvements should be coupled with social communication campaigns aimed at changing people's water storage behaviors.

*Social communication strategies.* Social mobilization and communication interventions should be developed to increase community members' dengue knowledge and, more importantly, to promote the adoption of preventative behaviors [63], [64]. A study conducted in these same areas found that community members have common misconceptions about dengue transmission and the mosquito vector, and community members identified lack of information as a barrier to taking actions to prevent dengue (Stewart Ibarra et al., *in prep*). Previous studies also found that lower dengue knowledge and lack of health education were associated with the presence of *Ae. aegypti* juveniles [17], [18]. Intuitively, the findings from this study suggest that dengue prevention messages should reflect seasonal changes in key larval habitats and neighborhood-specific risk factors. For example, public health messages at the beginning of the rainy season could focus on garbage disposal practices, whereas messages during the post-rainy dry season could focus on water storage practices, especially in the urban periphery.

*Early warning system.* The results of this and previous studies in this region are contributing to inter-institutional efforts in Ecuador to develop dengue prediction models and early warning systems (EWS) using climate and non-climate information [22], [65]. An online geospatial database (GIS) could be used to integrate real-time climate, vector, and dengue virus surveillance information with household census data to generate spatiotemporal predictions of dengue risk (e.g., seasonal risk maps). Information about adult female *Ae. aegypti* and dengue virus dynamics is not currently available, but could potentially become part of the Ministry of Health surveillance system, allowing for improved predictions of dengue risk. These predictions would ideally provide the public health sector with increased lead-time to implement the vector control interventions described above, preventing dengue outbreaks more effectively.

## Supporting Information

Figure S1 **Time series of climate and ovitrap data.** Climate data used to predict *Aedes aegypti* oviposition dynamic in Machala, Ecuador (October 2010– June 2011): (A) Mean, maximum and minimum temperature (°C), (B) log of daily precipitation (mm/day), (C) relative humidity (%), and (D) ovitrap data (eggs/ovitrap/week) from the central area (CA), the peripheral area (PA), and both localities combined.
(TIF)

Figure S2 **Cross correlation plots for ovitrap and climate data.** Lagged correlation coefficient (0–19 weeks) between ovitrap abundance data for both localities combined (eggs/ovitrap/week) with (A) minimum temperature and (B) daily rainfall.
(TIF)

Figure S3 **Scatter plots for best-fit models versus observed ovitrap data.** Results of the best-fit models developed using ovitrap data from (A) both localities combined, (B) peripheral area (PA) only, and (C) central area (CA) only.
(TIF)

Table S1 Correlation matrix for the characteristics of containers positive for *Aedes aegypti* pupae (n = 62).
(DOC)

Table S2 Coding of correct and incorrect knowledge of dengue transmission and *Aedes aegypti* juvenile habitat from household surveys in the peripheral area (PA) and central area (CA).
(DOC)

Table S3 Correlations matrices for suites of household parameters tested in logistic regression models to predict the presence of *Aedes aegypti* pupae.
(DOC)

Table S4 Slope coefficient estimates and adjusted odds ratios (OR) with 95% confidence intervals (CI) for parameters included in the top-ranked logistic regression models for each suite of parameters to predict households positive for *Aedes aegypti* pupae in each season.
(DOC)

Table S5 Pupal surveys of *Aedes aegypti* and ovitrap data (3 week average) in peripheral (PA) and central areas (CA).
(DOC)

Table S6 Key breeding containers. The number of containers, proportion of positive containers, pupae productivity (pupae per container), and the proportion of pupae per container type for each season. No pupae were collected from elevated water tanks.
(DOC)

Table S7 Correlations matrices for parameters included in the best-fit logistic models to predict households positive for *Aedes aegypti* pupae for each season.
(DOC)

Table S8 Top competing models (Δ AICc <2) to predict the presence of *Aedes aegypti* pupae. Significant parameters (*P*≤0.05) in bold.
(DOCX)

Table S9 Top ranked models (Δ AICc <2) to predict weekly ovitrap egg counts. Significant parameters (*P*≤0.05) in bold.
(DOCX)

Text S1 **Executive summary in Spanish.**
(DOCX)
