# Supplementary figures and images for: Dengue Vector Dynamics (Aedes aegypti) Influenced by Climate and Social Factors in Ecuador: Implications for Targeted Control
Source: PLoS One. 2013 Nov 12;8(11):e78263. doi: 10.1371/journal.pone.0078263 (PMC3855798; doi:10.1371/journal.pone.0078263)

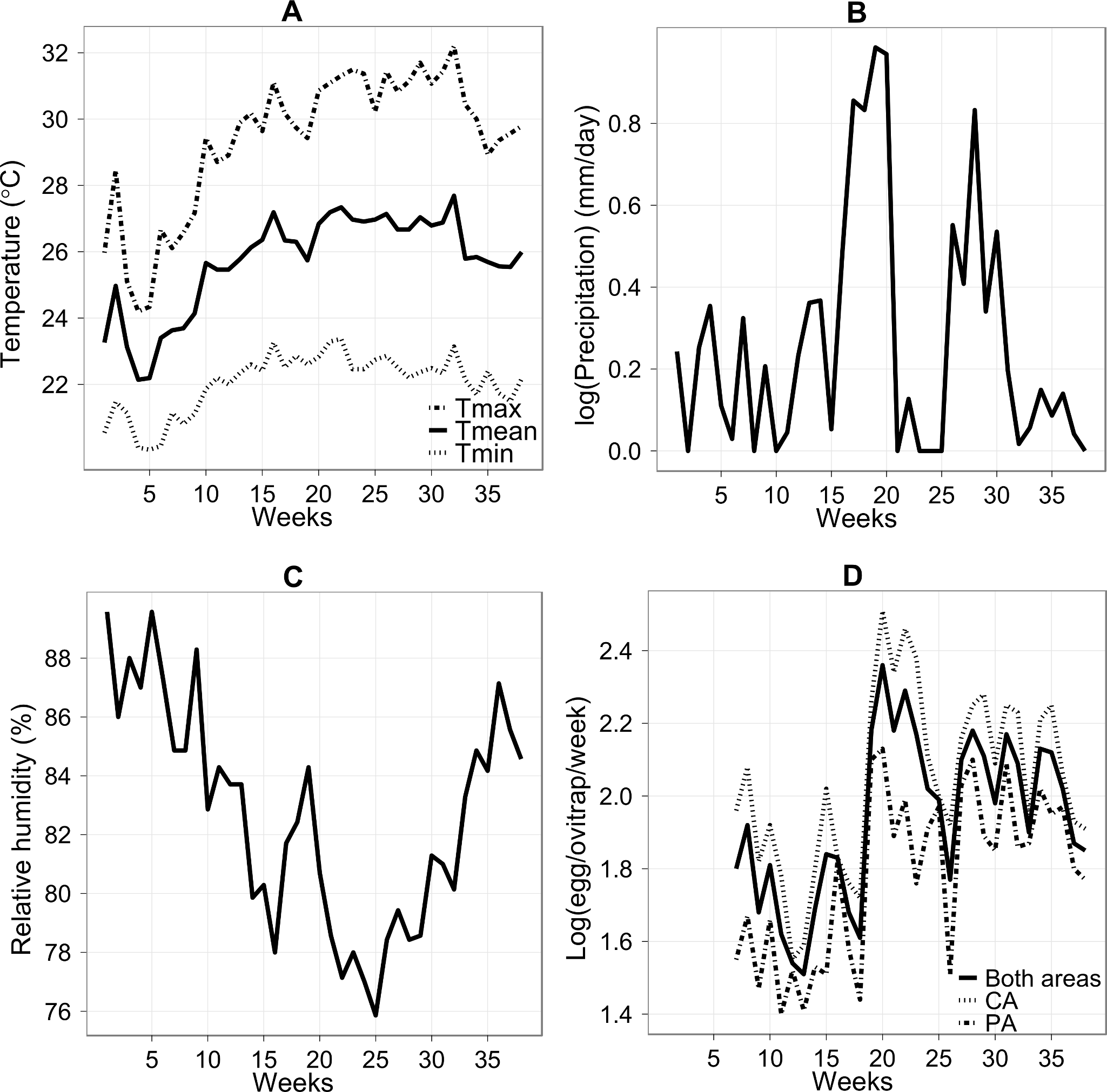

Supplement: Figure S1 — Time series of climate and ovitrap data. Climate data used to predict Aedes aegypti oviposition dynamic in Machala, Ecuador (October 2010– June 2011): (A) Mean, maximum and minimum temperature (°C), (B) log of daily precipitation (mm/day), (C) relative humidity (%), and (D) ovitrap data (eggs/ovitrap/week) from the central area (CA), the peripheral area (PA), and both localities combined. (TIF) [file pone.0078263.s001.tif]

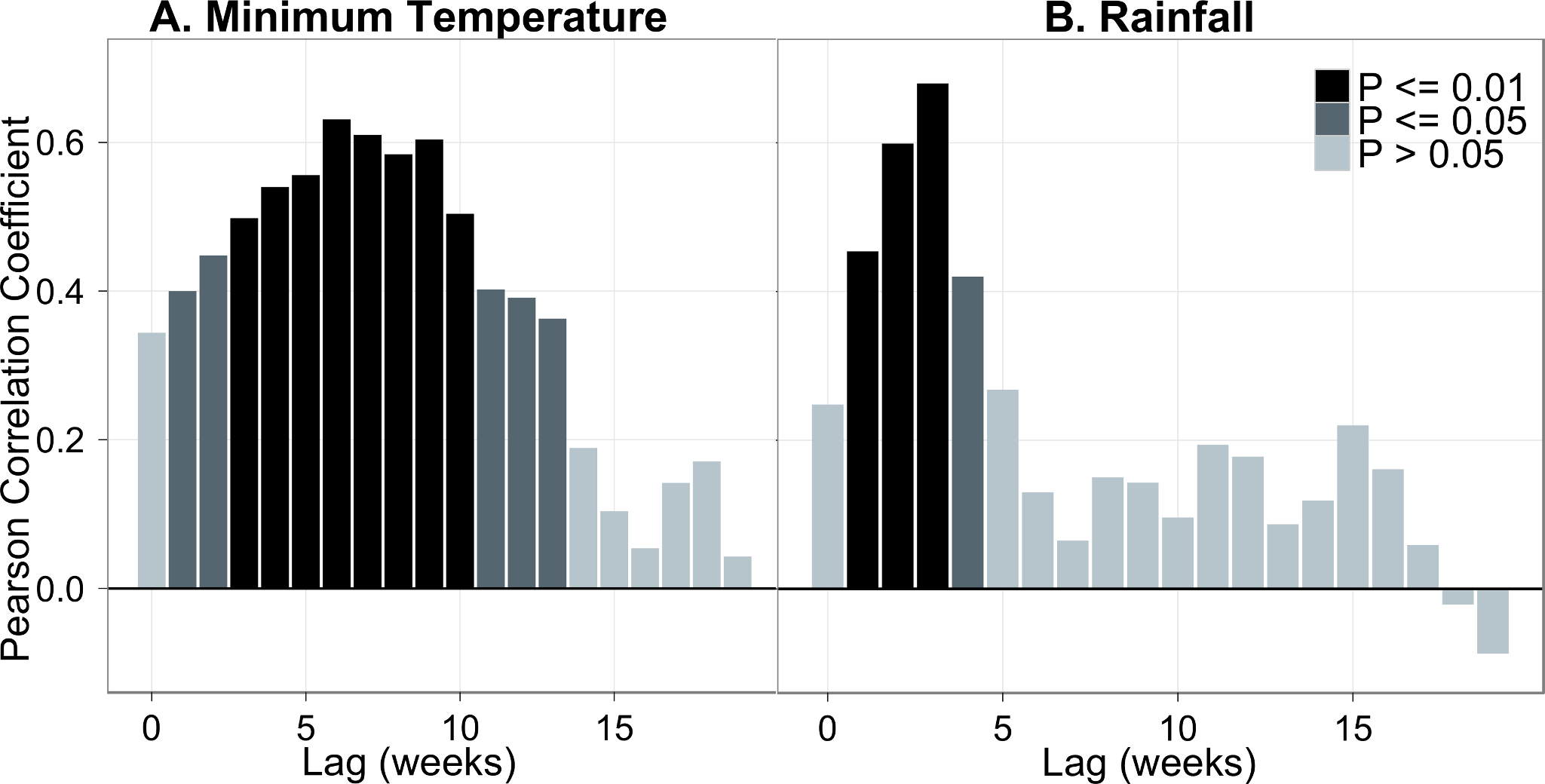

Supplement: Figure S2 — Cross correlation plots for ovitrap and climate data. Lagged correlation coefficient (0–19 weeks) between ovitrap abundance data for both localities combined (eggs/ovitrap/week) with (A) minimum temperature and (B) daily rainfall. (TIF) [file pone.0078263.s002.tif]

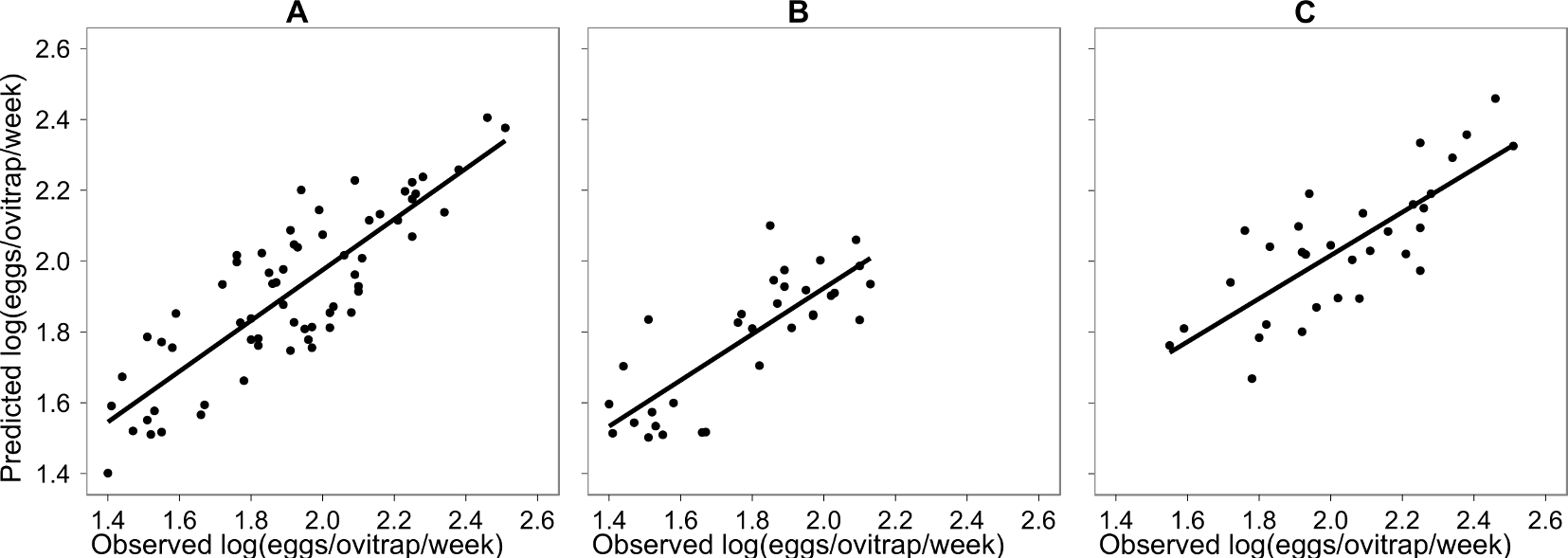

Supplement: Figure S3 — Scatter plots for best-fit models versus observed ovitrap data. Results of the best-fit models developed using ovitrap data from (A) both localities combined, (B) peripheral area (PA) only, and (C) central area (CA) only. (TIF) [file pone.0078263.s003.tif]
