# Supplementary material for: Dengue Vector Dynamics (Aedes aegypti) Influenced by Climate and Social Factors in Ecuador: Implications for Targeted Control
Source: PLoS One. 2013 Nov 12;8(11):e78263. doi: 10.1371/journal.pone.0078263 (PMC3855798; doi:10.1371/journal.pone.0078263)
Supplement: Table S1 — Correlation matrix for the characteristics of containers positive for Aedes aegypti pupae (n = 62). (DOC) [file pone.0078263.s004.doc]

| **Table S1.** Correlation matrix for the characteristics of containers positive for *Ae. aegypti* pupae (n = 62). | | | | | | | | | | | | |  |  |  |  |  |  | |  |  |  |
| --- | --- | --- | --- | --- | --- | --- | --- | --- | --- | --- | --- | --- | --- | --- | --- | --- | --- | --- | --- | --- | --- | --- |
|  |  |  | **Season** | | | **Container use** | | | | **Container type** | | | | | | **Water source** | | | **Location/shade** | | | |
|  | Log number of pupae | Central area | Pre-rain | Rain | Post-rain | Abandon | Decorative | Domestic | Animal water | 55 gal. barrel | Bucket | Med. Assort. | Small assort. | Vase | Tire | Tap | Rain | Inside | | Sunny | Shaded | Partial shade |
| Log number of pupae | 1 |  |  |  |  |  |  |  |  |  |  |  |  |  |  |  |  |  | |  |  |  |
| Central area | 0.10 | 1 |  |  |  |  |  |  |  |  |  |  |  |  |  |  |  |  | |  |  |  |
| **Season** |  |  |  |  |  |  |  |  |  |  |  |  |  |  |  |  |  |  | |  |  |  |
| Pre-rain | -0.03 | 0.24 | 1 |  |  |  |  |  |  |  |  |  |  |  |  |  |  |  | |  |  |  |
| Rain | 0.08 | 0.04 | -0.64** | 1 |  |  |  |  |  |  |  |  |  |  |  |  |  |  | |  |  |  |
| Post-rain | -0.06 | -0.32* | -0.34** | -0.50** | 1 |  |  |  |  |  |  |  |  |  |  |  |  |  | |  |  |  |
| **Container use** |  |  |  |  |  |  |  |  |  |  |  |  |  |  |  |  |  |  | |  |  |  |
| Abandoned | 0.21 | -0.04 | 0.12 | 0.13 | -0.29* | 1 |  |  |  |  |  |  |  |  |  |  |  |  | |  |  |  |
| Decorative | -0.27* | 0.23 | 0.02 | 0.06 | -0.10 | -0.36** | 1 |  |  |  |  |  |  |  |  |  |  |  | |  |  |  |
| Domestic | 0.02 | -0.18 | -0.17 | -0.17 | 0.40** | -0.70** | -0.33** | 1 |  |  |  |  |  |  |  |  |  |  | |  |  |  |
| Animal water | -0.11 | 0.15 | 0.08 | 0.01 | -0.09 | -0.16 | -0.08 | -0.15 | 1 |  |  |  |  |  |  |  |  |  | |  |  |  |
| **Container type** |  |  |  |  |  |  |  |  |  |  |  |  |  |  |  |  |  |  | |  |  |  |
| 55 gal. barrel | 0.35** | -0.02 | -0.01 | -0.12 | 0.15 | -0.33** | -0.28* | 0.59** | -0.13 | 1 |  |  |  |  |  |  |  |  | |  |  |  |
| Bucket | -0.28* | -0.14 | 0.01 | -0.13 | 0.15 | 0.08 | -0.24 | 0.14 | -0.11 | -0.41** | 1 |  |  |  |  |  |  |  | |  |  |  |
| Med. Assort. | 0.15 | 0.04 | 0.02 | 0.12 | -0.17 | 0.26* | 0.02 | -0.26* | -0.06 | -0.23 | -0.19 | 1 |  |  |  |  |  |  | |  |  |  |
| Small assort. | -0.21 | 0.11 | 0.06 | 0.07 | -0.15 | -0.02 | 0.05 | -0.24 | 0.62** | -0.20 | -0.18 | -0.10 | 1 |  |  |  |  |  | |  |  |  |
| Vase | -0.21 | 0.18 | -0.02 | 0.06 | -0.06 | -0.31* | 0.87** | -0.28* | -0.07 | -0.25 | -0.21 | -0.12 | -0.11 | 1 |  |  |  |  | |  |  |  |
| Tire | 0.11 | -0.09 | -0.05 | 0.11 | -0.08 | 0.44** | -0.16 | -0.31* | -0.07 | -0.27* | -0.23 | -0.13 | -0.11 | -0.14 | 1 |  |  |  | |  |  |  |
| **Water source** |  |  |  |  |  |  |  |  |  |  |  |  |  |  |  |  |  |  | |  |  |  |
| Tap | -0.18 | 0.03 | -0.06 | -0.23 | 0.34** | -0.78** | 0.22 | 0.64** | -0.01 | 0.39** | -0.02 | -0.23 | -0.07 | 0.14 | -0.40** | 1 |  |  | |  |  |  |
| Rain | 0.13 | -0.05 | 0.08 | 0.26* | -0.40** | 0.74** | -0.20 | -0.61** | 0.01 | -0.37** | -0.04 | 0.24 | 0.08 | -0.13 | 0.41** | -0.97** | 1 |  | |  |  |  |
| **Location/shade** |  |  |  |  |  |  |  |  |  |  |  |  |  |  |  |  |  |  | |  |  |  |
| Inside the home | 0.00 | -0.08 | -0.22 | 0.12 | 0.10 | -0.29* | 0.33** | 0.08 | -0.06 | 0.12 | -0.19 | -0.11 | -0.10 | 0.400** | -0.13 | 0.32* | -0.31* | 1 | |  |  |  |
| Sunny | 0.00 | 0.04 | 0.31* | -0.11 | -0.22 | 0.23 | -0.11 | -0.17 | 0.06 | -0.01 | -0.13 | 0.10 | 0.03 | -0.16 | 0.22 | -0.36** | 0.39** | -0.24 | | 1 |  |  |
| Shaded | -0.310* | 0.13 | -0.15 | 0.06 | 0.10 | -0.41** | 0.35** | 0.14 | 0.05 | -0.17 | 0.16 | -0.03 | 0.02 | 0.359** | -0.30* | 0.48** | -0.45** | 0.43** | | -0.57** | 1 |  |
| Partial shade | 0.33** | -0.18 | -0.17 | 0.06 | 0.13 | 0.19 | -0.25* | 0.03 | -0.11 | 0.20 | -0.03 | -0.08 | -0.05 | -0.22 | 0.09 | -0.13 | 0.08 | -0.20 | | -0.46** | -0.47** | 1 |

*Significant correlation at *P* ≤ 0.05, ** *P* ≤ 0.01
