# Supplementary material for: Dengue Vector Dynamics (Aedes aegypti) Influenced by Climate and Social Factors in Ecuador: Implications for Targeted Control
Source: PLoS One. 2013 Nov 12;8(11):e78263. doi: 10.1371/journal.pone.0078263 (PMC3855798; doi:10.1371/journal.pone.0078263)
Supplement: Table S2 — Coding of correct and incorrect knowledge of dengue transmission and Aedes aegypti juvenile habitat from household surveys in the peripheral area (PA) and central area (CA). (DOC) [file pone.0078263.s005.doc]

| **Table S2. Coding of dengue knowledge from surveys** | | |
| --- | --- | --- |
|  | PA (n = 39) | CA (n = 40) |
|  | n (%) | n (%) |
| **Knowledge of *Ae. aegypti* juvenile habitat** | | |
| **Correct** |  |  |
| Water tanks | 2 (5) | 3 (8) |
| Plants, flower vase | 2 (5) | 2 (5) |
| Bottles | 4 (10) | 0 |
| Containers: other | 14 (36) | 7 (18) |
| Standing water | 5 (13) | 15 (38) |
| Water (general) | 8 (21) | 6 (15) |
| Tires | 6 (15) | 7 (18) |
| Clean water | 5 (13) | 4 (10) |
| **Incorrect** |  |  |
| Dirty water or sewerage | 2 (5) | 4 (10) |
| Puddles | 10 (26) | 11 (28) |
| Other | 1 (3) | 1 (3) |
| **Knowledge of dengue transmission** | | |
| **Correct** |  |  |
| Mosquitoes | 27 (69) | 31 (78) |
| **Incorrect** |  |  |
| Water | 7 (18) | 7 (18) |
| Direct contact | 0 | 1 (3) |
| Dirty environment | 0 | 1 (3) |
| Don't know | 12 (31) | 9 (23) |

Coding of correct and incorrect knowledge of dengue transmission and *Ae. aegypti* juvenile habitat from household surveys in the peripheral study area (PA) and central area (CA).
