# Supplementary material for: Dengue Vector Dynamics (Aedes aegypti) Influenced by Climate and Social Factors in Ecuador: Implications for Targeted Control
Source: PLoS One. 2013 Nov 12;8(11):e78263. doi: 10.1371/journal.pone.0078263 (PMC3855798; doi:10.1371/journal.pone.0078263)
Supplement: Table S3 — Correlations matrices for suites of household parameters tested in logistic regression models to predict the presence of Aedes aegypti pupae. (DOC) [file pone.0078263.s006.doc]

**Table S3.** Correlations matrices for suites of household parameters tested in logistic regression models to predict the presence of *Aedes aegypti* pupae.

| **1. Demographics** |  |  |  |  |  |  |  |  |  |  |
| --- | --- | --- | --- | --- | --- | --- | --- | --- | --- | --- |
|  | Female head | Post secondary | Num. households | People per room | Total people | Renters | Young family | Older family | Old family | Employed |
| Female head | 1 |  |  |  |  |  |  |  |  |  |
| Post secondary | -0.005 | 1 |  |  |  |  |  |  |  |  |
| Num. households | 0.221* | -0.049 | 1 |  |  |  |  |  |  |  |
| People per room | -0.102 | -0.199 | -0.126 | 1 |  |  |  |  |  |  |
| Total people | -0.302** | -0.188 | -0.182 | 0.532** | 1 |  |  |  |  |  |
| Renters | -0.005 | 0.164 | 0.345** | -0.128 | -0.091 | 1 |  |  |  |  |
| Young family | -0.054 | -0.156 | -0.051 | 0.378** | 0.464** | -0.073 | 1 |  |  |  |
| Older family | 0.002 | 0.233* | -0.064 | -0.302** | -0.321** | 0.041 | -0.893** | 1 |  |  |
| Old family | 0.114 | -0.146 | 0.249* | -0.191 | -0.338** | 0.074 | -0.303** | -0.157 | 1 |  |
| Employed | -0.133 | 0.02 | 0.067 | 0.009 | 0.154 | 0.029 | 0.14 | -0.099 | -0.099 | 1 |

| **2. Wateraccess and storage** | |  |  |  |  |
| --- | --- | --- | --- | --- | --- |
|  | Piped water inside | Constant piped water | No cist/ET & do store | Cist/ET & don't store | Cist/ET & do store |
| Piped water inside | 1 |  |  |  |  |
| Constant piped water | 0.444** | 1 |  |  |  |
| No cist/ET & do store | -0.539** | -0.581** | 1 |  |  |
| Cist/ET &don'tstore | 0.664** | 0.521** | -0.633** | 1 |  |
| Cist/ET & do store | -0.281* | -0.058 | -0.232* | -0.606** | 1 |

| **3. Knowledge and perceptions** |  |  |  |  |  |
| --- | --- | --- | --- | --- | --- |
|  | Knowledge: Mosquito habitat | Knowledge: transmission | Dengue is a problem | Dengue is severe | Dengue is preventable |
| Knowledge: mosquito habitat | 1 |  |  |  |  |
| Knowledge: transmission | 0.382** | 1 |  |  |  |
| Dengue is a problem | -0.055 | 0.115 | 1 |  |  |
| Dengue is severe | -0.02 | 0.114 | 0.179 | 1 |  |
| Dengue is preventable | 0.162 | 0.216 | 0.06 | -0.001 | 1 |

| **4. Housing condition** | |  |  |  |  |  |  |  |  |  |  |  |  |
| --- | --- | --- | --- | --- | --- | --- | --- | --- | --- | --- | --- | --- | --- |
|  | Abandoned lots | Log patio area | Density of trees | Bad patio | Normal patio | Good patio | Bad house | Normal house | Good house | Low shade | Medium shade | High shade | No screens |
| Abandoned lots | 1 |  |  |  |  |  |  |  |  |  |  |  |  |
| Log patio area | 0.162 | 1 |  |  |  |  |  |  |  |  |  |  |  |
| Density of trees | 0.231* | -0.278* | 1 |  |  |  |  |  |  |  |  |  |  |
| Bad patio | 0.109 | 0.012 | 0.213 | 1 |  |  |  |  |  |  |  |  |  |
| Normal patio | -0.024 | 0.13 | -0.215 | -0.694** | 1 |  |  |  |  |  |  |  |  |
| Good patio | -0.103 | -0.181 | 0.015 | -0.334** | -0.447** | 1 |  |  |  |  |  |  |  |
| Bad house | 0.053 | -0.082 | 0.031 | 0.142 | -0.073 | -0.082 | 1 |  |  |  |  |  |  |
| Normal house | 0.025 | 0.106 | -0.07 | 0.043 | 0.054 | -0.125 | -0.377** | 1 |  |  |  |  |  |
| Good house | -0.068 | -0.034 | 0.041 | -0.159 | 0.008 | 0.187 | -0.467** | -0.643** | 1 |  |  |  |  |
| Low shade | -0.147 | -0.162 | -0.182 | 0.144 | -0.067 | -0.092 | -0.046 | 0.144 | -0.1 | 1 |  |  |  |
| Medium shade | 0.036 | 0.125 | 0.153 | -0.141 | 0.033 | 0.132 | 0.043 | -0.087 | 0.048 | -0.795** | 1 |  |  |
| High shade | 0.173 | 0.065 | 0.05 | -0.006 | 0.053 | -0.062 | 0.006 | -0.09 | 0.081 | -0.328** | -0.312** | 1 |  |
| No screens | -0.088 | 0.155 | -0.129 | 0.105 | 0.094 | -0.253* | 0.405** | 0.213 | -0.538** | 0.049 | 0.055 | -0.161 | 1 |

*Significant correlation at *P*≤ 0.05, ** *P*≤ 0.01
