# Supplementary material for: Dengue Vector Dynamics (Aedes aegypti) Influenced by Climate and Social Factors in Ecuador: Implications for Targeted Control
Source: PLoS One. 2013 Nov 12;8(11):e78263. doi: 10.1371/journal.pone.0078263 (PMC3855798; doi:10.1371/journal.pone.0078263)
Supplement: Table S4 — Slope coefficient estimates and adjusted odds ratios (OR) with 95% confidence intervals (CI) for parameters included in the top-ranked logistic regression models for each suite of parameters to predict households positive for Aedes aegypti pupae in each season. (DOC) [file pone.0078263.s007.doc]

| **Table S4.** Slope coefficient estimates and adjusted odds ratios (OR) with 95% confidence intervals (CI) for parameters included in the top-ranked logistic regression models for each suite of parameters to predict households positive for *Aedes aegypti* pupae in each season. | | | | | | |
| --- | --- | --- | --- | --- | --- | --- |
| **Parameters** | **β estimate** | **SE** | **OR** | **Lower 95% CI** | **Upper 95% CI** | ***P* value** |
| **A. Pre-rainy season (Nov 2010)** |  |  |  |  |  |  |
| **1. Demographics & SES** |  |  |  |  |  |  |
| Intercept | 0.07 | 1.13 |  |  |  | 0.95 |
| Female head of household | -1.96 | 1.24 | 0.14 | 0.01 | 1.61 | 0.12 |
| Old family | 2.06 | 1.82 | 7.88 | 0.22 | 280.03 | 0.26 |
| People per room in the household | -1.32 | 0.84 | 0.27 | 0.05 | 1.38 | 0.12 |
| Three or more households | 3.09 | 1.59 | 22.00 | 0.98 | 493.31 | 0.05 |
| Renters present on the property | -2.84 | 1.91 | 0.06 | 0.00 | 2.48 | 0.14 |
| **2. Water access/storage** |  |  |  |  |  |  |
| Intercept | -4.55 | 1.33 |  |  |  |  |
| Piped water inside the home | 2.76 | 1.28 | 15.76 | 1.28 | 194.81 | 0.02 |
| Cist/ET & do store water | 3.36 | 1.56 | 28.69 | 2.98 | 276.34 | < 0.01 |
| **3. Knowledge & attitude** |  |  |  |  |  |  |
| Intercept | -1.01 | 0.41 |  |  |  | 0.99 |
| Dengue is a severe disease | -1.13 | 0.67 | 0.32 | 0.09 | 1.2 | 0.09 |
| **4. House condition** |  |  |  |  |  |  |
| Intercept | -1.75 | 0.55 |  |  |  | < 0.01 |
| Bad patio condition | 1.73 | 0.75 | 5.62 | 1.29 | 24.54 | 0.02 |
| Patio is < 25% shaded | -1.88 | 0.88 | 0.15 | 0.03 | 0.86 | 0.03 |
| **B. Rainy season (Feb 2011)** |  |  |  |  |  |  |
| **1. Demographics & SES** |  |  |  |  |  |  |
| Intercept | -0.67 | 0.29 |  |  |  | 0.02 |
| Older family | -1.18 | 0.69 | 0.31 | 0.08 | 1.18 | 0.09 |
| **2. Water access/storeage** |  |  |  |  |  |  |
| Intercept | -1.23 | 0.30 |  |  |  | < 0.01 |
| Cist/ET & do store water | 1.52 | 0.62 | 4.57 | 1.36 | 15.4 | 0.01 |
| **3. Knowledge & attitude** |  |  |  |  |  |  |
| Intercept | 0.62 | 0.5921 |  |  |  | 0.29 |
| Knowledge of mosquito breeding | -1.41 | 0.59 | 0.24 | 0.08 | 0.78 | 0.02 |
| Dengue is a severe disease | -0.85 | 0.53 | 0.43 | 0.15 | 1.21 | 0.11 |
| **4. House condition** |  |  |  |  |  |  |
| Intercept | -1.78 | 0.42 |  |  |  | < 0.01 |
| Bad patio condition | 1.54 | 0.57 | 4.66 | 1.52 | 14.27 | 0.01 |
| Bad house condition | 1.30 | 0.64 | 3.65 | 1.05 | 12.69 | 0.04 |
| **C. Post rainy season (June 2011)** |  |  |  |  |  |  |
| **1. Demographics & SES** |  |  |  |  |  |  |
| Intercept | -0.64 | 0.41 |  |  |  | 0.70 |
| One household | -1.78 | 0.66 | 0.17 | 0.05 | 0.62 | < 0.01 |
| **2. Water access/storage** |  |  |  |  |  |  |
| Intercept | -0.76 | 0.55 |  |  |  | 0.17 |
| Constant access to piped water | -2.65 | 0.86 | 0.07 | 0.01 | 0.38 | < 0.01 |
| Cist/ET & do store water | 2.78 | 0.89 | 16.1 | 2.81 | 92.3 | < 0.01 |
| **3. Knowledge & attitude** |  |  |  |  |  |  |
| Intercept | -2.20 | 0.84 |  |  |  | 0.01 |
| Dengue is a problem | -2.20 | 0.84 | 0.11 | 0.02 | 0.58 | 0.01 |
| **4. House condition** |  |  |  |  |  |  |
| Intercept | -1.16 | 0.36 |  |  |  | < 0.01 |
| Good house condition | -1.07 | 0.71 | 0.34 | 0.09 | 1.37 | 0.13 |
