# Supplementary material for: Dengue Vector Dynamics (Aedes aegypti) Influenced by Climate and Social Factors in Ecuador: Implications for Targeted Control
Source: PLoS One. 2013 Nov 12;8(11):e78263. doi: 10.1371/journal.pone.0078263 (PMC3855798; doi:10.1371/journal.pone.0078263)
Supplement: Table S5 — Pupal surveys of Aedes aegypti and ovitrap data (3 week average) in peripheral (PA) and central areas (CA). (DOC) [file pone.0078263.s008.doc]

| **Table S5.** Pupal surveys of *Aedes aegypti* and ovitrap data (3 week average) in peripheral (PA) and central study areas (CA) in Machala, Ecuador | | | | | | | | | | |  |
| --- | --- | --- | --- | --- | --- | --- | --- | --- | --- | --- | --- |
|  |  | Households | | Containers | | | Pupae collected | | Ovitrap | |  |
| Survey | Site | N | Prop. with pupae | N | N with pupae | Prop. with pupae | N | Pupal index [mean (se)] | | Eggs per ovitrap [mean (se)] | |
| Pre rainy season | PA | 40 | 0.10 | 398 | 4 | 0.01 | 28 | 0.34 (0.31) | 36.42 (8.53) | |  |
| (Nov 15 - Dec 1, 2010) | CA | 37 | 0.30 | 577 | 15 | 0.03 | 261 | 2.10 (1.09) | 89.14 (18.58) | |  |
|  | Both sites | 77 | 0.19 | 975 | 19 | 0.02 | 289 | 1.17 (0.54) | 62.78 (10.58) | |  |
| Rainy season | PA | 40 | 0.23 | 409 | 11 | 0.03 | 96 | 0.89 (1.3) | 74.60 (10.44) | |  |
| (Feb 22 - March 11, 2011) | CA | 40 | 0.35 | 718 | 19 | 0.03 | 280 | 2.71 (0.61) | 238.36 (35.60) | |  |
|  | Both sites | 80 | 0.29 | 1127 | 30 | 0.03 | 376 | 1.80 (0.72) | 157.49 (20.75) | |  |
| Post-rainy season | PA | 39 | 0.23 | 184 | 9 | 0.05 | 98 | 0.69 (0.37) | 76.15 (14.42) | |  |
| (May 31 - June 13, 2011) | CA | 38 | 0.11 | 206 | 4 | 0.02 | 46 | 0.58 (0.55) | 111.21 (18.32) | |  |
|  | Both sites | 77 | 0.17 | 390 | 13 | 0.03 | 144 | 0.63 (0.33) | 93.68 (11.75) | |  |
