# Supplementary material for: Dengue Vector Dynamics (Aedes aegypti) Influenced by Climate and Social Factors in Ecuador: Implications for Targeted Control
Source: PLoS One. 2013 Nov 12;8(11):e78263. doi: 10.1371/journal.pone.0078263 (PMC3855798; doi:10.1371/journal.pone.0078263)
Supplement: Table S6 — Key breeding containers. The number of containers, proportion of positive containers, pupae productivity (pupae per container), and the proportion of pupae per container type for each season. No pupae were collected from elevated water tanks. (DOC) [file pone.0078263.s009.doc]

| **Table S6.** Key breeding containers. The number of containers, proportion of positive containers, pupae productivity (pupae per container), and the proportion of pupae per container type for each season in Machala, Ecuador. No pupae were collected from elevated water tanks. | | | | | | | | | | | | |
| --- | --- | --- | --- | --- | --- | --- | --- | --- | --- | --- | --- | --- |
|  | Pre-rainy season | | | | Rainy season | | | | Post-rainy season | | | |
| Container type | N | Prop. positive | Productivity [mean (se)] | Prop. of pupae | N | Prop. positive | Productivity [mean (se)] | Prop. of pupae | N | Prop. positive | Productivity [mean (se)] | Prop. of pupae |
| Barrel | 63 | 0.10 | 33.0 (20.68) | 0.69 | 67 | 0.12 | 17.75 (7.64) | 0.38 | 37 | 0.16 | 15.17 (7.54) | 0.63 |
| Medium assort. | 57 | 0.04 | 20.5 (5.63) | 0.14 | 48 | 0.08 | 28.0 (15.5) | 0.30 | 15 | 0.00 | 0.00 | 0.00 |
| Tire | 7 | 0.29 | 12.0 (6.20) | 0.08 | 17 | 0.29 | 13.4 (11.77) | 0.18 | 4 | 0.25 | 6.0 (3.46) | 0.04 |
| Bucket & wash basin | 329 | 0.02 | 4.2 (1.06) | 0.07 | 236 | 0.03 | 3.67 (0.91) | 0.06 | 145 | 0.03 | 9.2 (4.85) | 0.32 |
| Vase | 26 | 0.08 | 1.0 (0.32) | 0.01 | 30 | 0.13 | 6.0 (3.26) | 0.06 | 13 | 0.08 | 1.0 (0.32) | 0.01 |
| Small assort. | 106 | 0.02 | 1.5 (0.27) | 0.01 | 148 | 0.02 | 3.0 (0.54) | 0.02 | 71 | 0.00 | 0.00 | 0.00 |
| Cistern | 30 | 0.01 | 0.03 (0.03) | .003 | 33 | 0.00 | 0.0 | 0.00 | 34 | 0.00 | 0.00 | 0.00 |
