# Supplementary material for: Dengue Vector Dynamics (Aedes aegypti) Influenced by Climate and Social Factors in Ecuador: Implications for Targeted Control
Source: PLoS One. 2013 Nov 12;8(11):e78263. doi: 10.1371/journal.pone.0078263 (PMC3855798; doi:10.1371/journal.pone.0078263)
Supplement: Table S7 — Correlations matrices for parameters included in the best-fit logistic models to predict households positive for Aedes aegypti pupae for each season. (DOC) [file pone.0078263.s010.doc]

**Table S7.** Correlations matrices for parameters included in the best-fit logistic models to predict households positive for *Aedes aegypti* pupae for each season.

| **Pre-rainy season** |  |  |  |  |  |  |  |
| --- | --- | --- | --- | --- | --- | --- | --- |
|  | Pupae | Renters | 3+ families | Old family | Piped water inside | Cist/ET & do store | Bad patio |
| Pupae | 1 |  |  |  |  |  |  |
| Renters | -0.086 | 1 |  |  |  |  |  |
| 3+ families | 0.273* | 0.326** | 1 |  |  |  |  |
| Old family | 0.275* | -0.091 | 0.126 | 1 |  |  |  |
| Piped water inside | 0.148 | 0.102 | -0.017 | 0.146 | 1 |  |  |
| Cist/ET & do store | 0.361** | 0.112 | 0.028 | -0.106 | -0.319** | 1 |  |
| Bad patio | 0.300* | -0.051 | -0.02 | 0.139 | -0.166 | 0.263* | 1 |
| **Rainy season** |  |  |  |  |  |  |  |
|  | Pupae | Central area | Knowledge: Mosquito habitat | Cist/ET & do store | Older family | Bad house | Bad patio |
| Pupae | 1 |  |  |  |  |  |  |
| Central area | 0.116 | 1 |  |  |  |  |  |
| Knowledge: Mosquito habitat | -0.240* | 0.129 | 1 |  |  |  |  |
| Cist/ET & do store | 0.295** | 0 | -0.088 | 1 |  |  |  |
| Older family | -0.168 | 0.2 | -0.081 | 0.056 | 1 |  |  |
| Bad house | 0.284* | -0.032 | 0.045 | 0.071 | -0.216 | 1 |  |
| Bad patio | 0.356** | 0.084 | -0.119 | 0.245* | -0.034 | 0.162 | 1 |
| **Post-rainy season** |  |  |  |  |  |  |  |
|  | Pupae | Dengue is a problem | One family | Constant piped water | Cist/ET & do store |  |  |
| Pupae | 1 |  |  |  |  |  |  |
| Dengue is a problem | -0.339** | 1 |  |  |  |  |  |
| One family | -0.335** | 0.058 | 1 |  |  |  |  |
| Constant piped water | -0.404** | 0.358** | -0.01 | 1 |  |  |  |
| Cist/ET & do store | 0.415** | 0.034 | -0.087 | -0.055 | 1 |  |  |
|  |  |  |  |  |  |  |  |
| *Significant correlation at the P < 0.05 level; ** at the P < 0.01 level | | | | |  |  |  |
