# Supplementary material for: Dengue Vector Dynamics (Aedes aegypti) Influenced by Climate and Social Factors in Ecuador: Implications for Targeted Control
Source: PLoS One. 2013 Nov 12;8(11):e78263. doi: 10.1371/journal.pone.0078263 (PMC3855798; doi:10.1371/journal.pone.0078263)
Supplement: Table S8 — Top competing models (Δ AICc <2) to predict the presence of Aedes aegypti pupae. Significant parameters (P≤0.05) in bold. (DOCX) [file pone.0078263.s011.docx]

| **Table S8**. Top competing models (∆ AICc < 2) to predict the presence of *Aedes aegypti* pupae. Significant parameters (*P* ≤ 0.05) in bold. | | | | | | |
| --- | --- | --- | --- | --- | --- | --- |
| **Pre-rainy season** | | | AICc | | Weight | ∆ AICc |
| 1 | Pupae1 ~ 1 + **renters + family3** + old_fam + **piped_inside + store3 + badpatio** | | 41.911 | | 0.058 | 0.000 |
| 2 | Pupae1 ~ 1 + fem_head + renters + **family3** + old_fam + **piped_inside** + lowshade + **store3** + badpatio | | 42.149 | | 0.052 | 0.238 |
| 3 | Pupae1 ~ 1 + fem_head + renters + **family3** + old_fam + **piped_inside + store3** + badpatio | | 42.695 | | 0.039 | 0.785 |
| 4 | Pupae1 ~ 1 + **renters + family3 + piped_inside + store3 + badpatio** | | 42.782 | | 0.038 | 0.871 |
| 5 | Pupae1 ~ 1 + **renters + family3 + piped_inside** + lowshade + **store3 + badpatio** | | 42.928 | | 0.035 | 1.017 |
| 6 | Pupae1 ~ 1 + renters + **family3** + old_fam + **piped_inside** + lowshade + **store3 + badpatio** | | 42.929 | | 0.035 | 1.018 |
| 7 | Pupae1 ~ 1 + fem_head + renters + **family3 + piped_inside** + lowshade + **store3 + badpatio** | | 42.958 | | 0.034 | 1.047 |
| 8 | Pupae1 ~ 1 + renters + **family3 +** old_fam + **piped_inside + store3** | | 43.619 | | 0.025 | 1.709 |
| 9 | Pupae1 ~ 1 + fem_head + pplrm + **renters + family3** + old_fam + **piped_inside** + lowshade + **store3** + badpatio | | 43.839 | | 0.022 | 1.928 |
| **Rainy season** | | |  | |  |  |
| 1 | Pupae2 ~ 1 + **mosq_right** + older_fam + **store3 + badpatio + badhouse** + central_area | | 85.475 | | 0.056 | 0.000 |
| 2 | Pupae 2 ~ 1 + **mosq_right** + store3 + **badpatio + badhouse** | | 85.525 | | 0.055 | 0.050 |
| 3 | Pupae2 ~ 1 + **mosq_right** + grave + **store3 + badpatio + badhouse** | | 85.561 | | 0.054 | 0.086 |
| 4 | Pupae2 ~ 1 + **mosq_right** + older_fam + **store3 + badpatio + badhouse** | | 85.918 | | 0.045 | 0.443 |
| 5 | Pupae2 ~ 1 + **mosq_right** + store3 + **badpatio + badhouse** + central_area | | 86.152 | | 0.040 | 0.677 |
| 6 | Pupae2 ~ 1 + **mosq_right** + grave + older_fam + **store3 + badpatio** + badhouse | | 86.299 | | 0.037 | 0.824 |
| 7 | Pupae2 ~ 1 + **mosq_right** + grave + older_fam + **store3** + badpatio + badhouse + central_area | | 86.386 | | 0.036 | 0.911 |
| 8 | Pupae2 ~ 1 + **mosq_right** + grave + **store3 + badpatio + badhouse** + central_area | | 86.577 | | 0.032 | 1.102 |
| 9 | Pupae2 ~ 1 + **mosq_right + badpatio + badhouse** | | 86.960 | | 0.027 | 1.485 |
| 10 | Pupae2 ~ 1 + **store3 + badpatio + badhouse** | | 87.240 | | 0.023 | 1.765 |
| 11 | Pupae2 ~ 1 + **mosq_right + older_fam + store3 + badpatio** + central_area | | 87.347 | | 0.022 | 1.872 |
| **Post-rainy season** | | |  | |  |  |
| 1 | Pupae3 ~ 1 + deng_problem + **family1 + alwayswater + store3** | | 34.147 | | 0.390 | 0.000 |
| 2 | Pupae3 ~ 1 + **deng_problem + family1 + alwayswater + store3** + goodhouse | | 35.454 | | 0.203 | 1.307 |
| 3 | Pupae3 ~ 1 + deng_problem + **family1 + alwayswater + store3** + central_area | | 35.927 | | 0.160 | 1.780 |
| **Parameter** | | **Abbreviation** | |  |  |  |
| Female head of household | | fem_head | |  |  |  |
| Average age of the family 35-64 | | older_fam | |  |  |  |
| Average age of the family >65 | | old_fam | |  |  |  |
| One independent household residing on the property | | family1 | |  |  |  |
| Three or more independent households residing on the property | | family3 | |  |  |  |
| Renters present on the property | | renters | |  |  |  |
| Piped water inside the home | | piped_inside | |  |  |  |
| Constant access to the household piped water supply | | alwayswater | |  |  |  |
| Water storage: Cist/ET & do store | | store3 | |  |  |  |
| Knowledge of mosquito habitat | | mosq_right | |  |  |  |
| Dengue is a problem | | deng_problem | |  |  |  |
| Low proportion of the patio area shaded | | lowshade | |  |  |  |
| Bad patio condition | | Badpatio | |  |  |  |
| Bad house condition | | badhouse | |  |  |  |
| Good house condition | | goodhouse | |  |  |  |
| Household located in the central area | | central_area | |  |  |  |
