# Supplementary material for: Dengue Vector Dynamics (Aedes aegypti) Influenced by Climate and Social Factors in Ecuador: Implications for Targeted Control
Source: PLoS One. 2013 Nov 12;8(11):e78263. doi: 10.1371/journal.pone.0078263 (PMC3855798; doi:10.1371/journal.pone.0078263)
Supplement: Table S9 — Top ranked models (Δ AICc <2) to predict weekly ovitrap egg counts. Significant parameters (P≤0.05) in bold. (DOCX) [file pone.0078263.s012.docx]

| **Table S9.** Top ranked models (∆ AICc < 2) to predict weekly ovitrap egg counts. Significant parameters (*P* ≤ 0.05) in bold. | | | | |
| --- | --- | --- | --- | --- |
|  | | **AICc** | **Weights** | **∆ AICc** |
| *Both sites combined* | |  |  |  |
| 1 | egglog ~ 1 + **prcplog + tempmin + relhum + tempmax + tempmean + site** | -49.05 | 0.20 | 0.00 |
| 2 | egglog ~ 1 + **prcplog + tempmin** + relhum + **site** | -48.57 | 0.16 | 0.48 |
| 3 | egglog ~ 1 + **prcplog + tempmin + site** | -48.18 | 0.13 | 0.87 |
| 4 | egglog ~ 1 + **prcplog + tempmin** + tempmax + **site** | -47.10 | 0.07 | 1.96 |
| *Peripheral area* | |  |  |  |
| 1 | egglog ~ 1 + prcplog + **tempmin** + relhum | -27.88 | 0.12 | 0.00 |
| 2 | egglog ~ 1 + prcplog + **tempmin** + tempmax | -27.64 | 0.11 | 0.23 |
| 3 | egglog ~ 1 + prcplog + **tempmin** + tempmean | -27.51 | 0.10 | 0.37 |
| 4 | egglog ~ 1 + **prcplog** + **tempmin** | -27.30 | 0.09 | 0.58 |
| 5 | egglog ~ 1 + **tempmin + relhum** | -27.14 | 0.08 | 0.74 |
| 6 | egglog ~ 1 + prcplog + **tempmin** + relhum + tempmax | -26.28 | 0.06 | 1.59 |
| 7 | egglog ~ 1 + prcplog + **tempmin** + relhum + tempmean | -26.12 | 0.05 | 1.76 |
| *Central area* | |  |  |  |
| 1 | egglog ~ 1 + **prcplog + tempmin** | -21.11 | 0.29 | 0.00 |
| 2 | egglog ~ 1 + **prcplog + tempmean** | -19.56 | 0.13 | 1.55 |
