# Supplementary material for: Dengue Vector Dynamics (Aedes aegypti) Influenced by Climate and Social Factors in Ecuador: Implications for Targeted Control
Source: PLoS One. 2013 Nov 12;8(11):e78263. doi: 10.1371/journal.pone.0078263 (PMC3855798; doi:10.1371/journal.pone.0078263)
Supplement: Text S1 — Executive summary in Spanish. (DOCX) [file pone.0078263.s013.docx]

**Text S1: Executive summary in Spanish**

**La dinámica del vector de dengue (*Aedes aegypti*) está influenciado por factores climáticos y sociales en la costa sur de Ecuador: Implicaciones para un control focalizado.**

**Introducción/Métodos:** Dengue, una enfermedad viral transmitida por mosquitos, es ahora la enfermedad tropical de propagación más rápida que ocurre a nivel mundial. Estudios previos indican que la interacción entre el clima y el comportamiento humano influencian en la dinámica del dengue, el virus y el vector (*Aedes aegypti*), sin embargo, efectos relativos de estas variables dependen de la ecología local y el contexto social. En este estudio, hemos investigado la influencia combinada del clima y de los factores socio-ecológicos en la dinámica de *Ae. aegypti* en Machala (Sur-oeste de Ecuador), seis meses después de la más grave epidemia de dengue registrada hasta la presente fecha. Se realizó un estudio longitudinal de *Ae. aegypti* en 80 hogares en dos áreas urbanas: un área central con acceso a los servicios públicos y un área periférica con acceso a limitado a servicios básicos. La ovoposición del mosquito fue monitoreada cada semana (desde noviembre 2010 a junio 2011); las encuestas de pupa fueron realizadas antes, durante y después de la temporada lluviosa para identificar los criaderos más importantes. Se condujeron encuestas de hogares para evaluar las características de vivienda, demografía, y comportamiento humano asociado con la presencia de *Ae. aegypti*.

**Resultados**: Los resultados de este estudio proveen evidencia de que la influencia del clima sobre la dinámica de *Ae. aegypti* varía según la estación lluviosa y el barrio en función de los factores socio-ecológicos locales.

1. En todo el periodo del estudio los recipientes abandonados y de uso doméstico contenían cantidades iguales de pupas de *Ae. aegypti,* aunque los recipientes abandonados contenían una proporción mayor de pupas durante la época de lluvias (65% de todas las pupas colectadas) y los recipientes de uso doméstico contenían una proporción mayor de pupas durante la época seca (66% de todas las pupas colectadas).
2. Se encontró que *Ae. aegypti* era más abundante en la zona central, donde recipientes abandonados y llenos de agua de lluvia (e.j., llantas, latas de comida) contenían mayor numero de pupa. Recipientes de uso doméstico (e.j., barriles) llenos con agua de la llave eran los criaderos con mayor numero de pupa la periferia. Los barriles en ambas áreas fueron los recipientes más productivos (contenían 77% de la pupa en la periferia; 44% de la pupa en la zona central), especialmente durante la época seca (63% de todas las pupas). Hemos encontrado que la dinámica del *Ae. aegypti* fue influenciada por la lluvia (3 semanas antes) y por la temperatura mínima (6 semanas antes); aunque la lluvia no influyó la reproducción de*Ae. aegyti*en la periferia, donde los criaderos principales fueron los recipientes de uso doméstico.
3. Se encontró que las pupas se concentraron en una pequeña proporción en los hogares: 11% de los hogares contenían 82% de las pupas durante la temporada de lluvia. Durante la temporada lluviosa, factores de riesgo para la presencia de pupas fueron, casas y patios en mal estado (e.j., áreas desordenadas y con basura), así como la falta de conocimiento de los lugares idóneos que *Ae. aegypti* usa como criadero. Durante la temporada seca, los hogares tenían mayor factor de riesgo si tuvieron interrupciones en el suministro de agua potable, si compartían su propiedad con dos o más familias, y si la gente sentía que el dengue no era un problema. En todas las estaciones, los hogares tenían un mayor factor riesgo, si tenían una cisterna o un tanque de agua elevado, así como almacenamiento de agua en recipientes bajos.

**Implicaciones para la salud pública:** Este estudio inicial sobre los factores de riesgo de dengue provee información que puede ser utilizada por las organizaciones regionales de salud pública para implementar campañas de control vectorial focalizados por temporadas (lluviosas y secas) en áreas urbanas.

1. Debido a que las pupas se concentraron en pocos hogares, encuestas rápidas de hogares podrían ser desarrolladas para identificar los hogares de alto riesgo y de esta forma sean objeto de control de los vectores en cada temporada.
2. Recomendamos el uso de encuestas pupales para identificar los tipos de recipientesmás productivos en cada temporada y zona urbana. Por ejemplo, en este estudio, el riesgo de dengue podría ser considerablemente reducido si los barriles fueran eliminados como sitios de criaderos para mosquitos (e.j, el uso de tapas y larvicida), especialmente en la periferia urbana, donde los barriles contenían 79% de todas las pupas.
3. Mejorar el acceso al suministro de agua potable en la periferia urbana puede reducir el riesgo de dengue, ya que la gente tendría menos necesidad de almacenar agua. Sin embargo, sin campañas de educación y comunicación, las personas podrían seguir almacenando agua a pesar de las mejoras al acceso del suministro de agua.
4. Se recomienda el uso demovilización social y estrategias de comunicación para aumentar el conocimiento de la gente sobre la transmisión del dengue y los hábitats de los vectores, así como promover la adopción de conductas (o actitudes) preventivas (por ejemplo, hábitos correctosde almacenamiento de agua), según lo recomendado por la OPS.
5. Estos resultados indican el potencial para desarrollar un sistema de alerta temprana para dengue utilizando información climática. Una base de datos geoespacial (GIS) en línea podría ser utilizada para integrar variables en tiempo real climático, vector e información de vigilancia del virus de dengue con los datos de los hogares obtenidos del censo para generar predicciones espaciotemporales del riesgo del dengue (por ejemplo, mapas de riesgo estacionales). Información sobre hembras adultas de *Ae. . aegypti* y dinámica del virus del dengue no está disponible actualmente, pero podría convertirse en parte del sistema de vigilancia del Ministerio de Salud, permitiendo mejorar las predicciones del riesgo de dengue.
